# Reprogramming of tumor-associated macrophages via NEDD4-mediated CSF1R degradation by targeting USP18

**DOI:** 10.1016/j.celrep.2023.113560

**Published:** 2023-12-13

**Authors:** Sayuri Miyauchi, Kei-ichiro Arimoto, Mengdan Liu, Yue Zhang, Dong-Er Zhang

**Affiliations:** 1 Moores Cancer Center, University of California San Diego, La Jolla, CA 92037, USA; 2 School of Biological Sciences, University of California San Diego, La Jolla, CA 92037, USA; 3 Department of Pathology, University of California San Diego, La Jolla, CA 92037, USA; 4 Lead contact

## Abstract

Tumor-associated myeloid cells modulate the tumor microenvironment and affect tumor progression. Type I interferon (IFN-I) has multiple effects on tumors and immune response, and ubiquitin-specific peptidase 18 (USP18) functions as a negative regulator of IFN-I signal transduction. This study aims to examine the function of IFN-I in myeloid cells during tumor progression. Here, we show that deletion of USP18 in myeloid cells suppresses tumor progression. Enhanced IFN-I signaling and blocked USP18 expression prompt downregulation of colony stimulating factor 1 receptor (CSF1R) and polarization of tumor-associated macrophages toward pro-inflammatory phenotypes. Further in vitro experiments reveal that downregulation of CSF1R is mediated by ubiquitin-proteasome degradation via E3 ligase neural precursor cell-expressed, developmentaly downregulated 4 (NEDD4) and the IFN-induced increase in ubiquitin E2 ubiquitin-conjugating enzyme H5. USP18 impairs ubiquitination and subsequent degradation of CSF1R by interrupting NEDD4 binding to CSF1R. These results reveal a previously unappreciated role of IFN-I in macrophage polarization by regulating CSF1R via USP18 and suggest targeting USP18 in myeloid-lineage cells as an effective strategy for IFN-based therapies.

## In brief

Miyauchi et al. report that deletion of USP18 in myeloid cells suppresses tumor progression by downregulation of CSF1R and increase in anti-tumor macrophages. USP18 impairs degradation of CSF1R by interrupting E3 ligase NEDD4 binding to CSF1R. These results reveal roles of IFN-I in macrophage polarization via the CSF1R-USP18 axis.

## Graphical Abstract

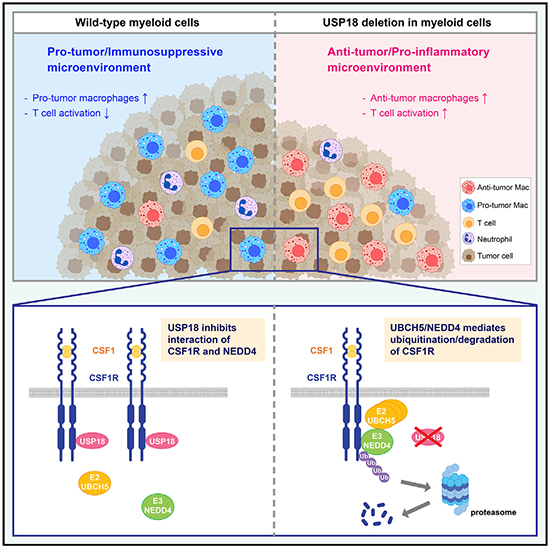

## INTRODUCTION

Accumulating evidence reveals that the tumor microenvironment (TME) has a fundamental effect on tumor development, growth, and therapeutic outcome. The TME is a complex network containing cytokines, chemokines, extracellular matrix, endothelial cells, fibroblasts, and immune cells. The constant interaction and communication among these cells and tumor cells support cancer development. Therefore, it is important to understand the molecular basis of establishment of the pro-cancer TME and to identify therapeutic interventions to modulate the TME for suppressing cancer development. Myeloid cells, including macrophages, dendritic cells (DCs), and granulocytes, are important in initiating both innate and adaptive immune responses as well as in supporting and inhibiting neoplasms.^1,2^ The most frequently identified non-tumor cells in the TME are tumor-associated macrophages (TAMs). They are considered immunosuppressive cells in the TME and promote tumor growth and metastasis by stimulation of matrix remodeling, angiogenesis, and secretion of growth factors and cytokines. Clinical data indicate that TAMs are generally associated with high tumor grade and poor prognosis in many human cancer types, such as cancers of the breast, bladder, prostate, and head and neck; glioma; melanoma; and non-Hodgkin’s lymphoma.^3–5^ However, increased TAMs have also been reported with better prognosis in colorectal and gastric cancers, suggesting that TAMs may have diversified functions in the TME. In fact, macrophages are known to have high plasticity and present different phenotypes in response to a variety of stimulations and environments. In response to the TME, TAMs are known to be polarized to either a pro-inflammatory/anti-tumorigenic phenotype or an immunosuppressive/pro-tumorigenic phenotype. Immunosuppressive macrophages produce anti-inflammatory cytokines and growth factors, such as IL-10 and TGF-β, creating a tumor-promoting microenvironment, whereas anti-tumor macrophages produce pro-inflammatory cytokines, such as TNF-α and IL-6.^6^ Importantly, most TAMs may have a spectrum of mixed phenotypes between the extreme anti-tumor and the pro-tumor phenotypes, and upon different stimulations, they are able to gain or lose those phenotypes.^5,7^ Therefore, it is significant to investigate how to reprogram TAMs toward the anti-tumor phenotypes to suppress tumor growth.

Type I interferons (IFN-Is) have direct effects on tumor cells, inhibiting their proliferation and inducing apoptosis.^8^ IFN-Is also have effects on immune cells through various mechanisms.^9^ IFN-Is support cytotoxic T lymphocytes (CTLs) by enhancing cross-priming from dendritic cells, boosting immune effector functions, and promoting their survival.^10,11^ IFN-Is also stimulate macrophages, leading to the release of pro-inflammatory cytokines, such as IL-1β and IL-18.^12^ Moreover, IFN-Is can decrease the immunosuppressive function of regulatory T cells.^13^ Further investigations are still required to understand the precise role of IFN-Is in anti-tumor immunity. In the past decades, IFN-I has been used for cancer treatment via systemic administration; however, there are limitations for clinical use due to the limited efficacy and the adverse effects such as fatigue, anorexia, flu-like symptoms, and hepatotoxicity. Thus, approaches to enhance effects but lower toxicity via targeted therapy and specific delivery of IFN-I should be considered.

Ubiquitin-specific peptidase 18 (USP18, aka UBP43) was first reported as a ubiquitin-like modifier ISG15-specific deconjugating enzyme and is responsible for removing ISG15 from ISGylated proteins.^14^ In addition to increased levels of ISG15-modified proteins, USP18-deficient cells have much stronger IFN-I signaling.^15^ Further studies reveal that USP18 is a potent negative regulator of IFN-I signaling via disruption of STAT2 binding to IFN-I receptor subunit R2 and blocking of JAK kinase activation.^16,17^ Deletion of USP18 enhances and prolongs IFN-I signaling^17,18^ and expands the pool of IFN-inducible genes.^19^ In addition to regulation of IFN-I signaling and protein ISG15 conjugation, USP18 is reported to regulate type III IFN signal transduction^20^ and expression of EGFR, CCND1, and other regulators in cancer cells.^21–23^ Altogether, USP18 regulates IFN signaling and cancer-related target gene expression, suggesting a potential, promising benefit of USP18-related studies for development of targeted anti-cancer therapies.

Despite its importance in the anti-tumor immune response, IFN-I in myeloid cells, especially in TAMs, is underinvestigated. Furthermore, there has been no report about the role of USP18 in TAMs. The aim of the current study is to examine the function of USP18 and IFN-I in macrophages during tumor development by utilizing myeloid-lineage-specific USP18-knockout (KO) mice. Here, we report that deletion of USP18 in myeloid cells suppresses tumor growth and enhances activation of cytotoxic CD8^+^ cells. This anti-tumor effect is induced by an increase in anti-tumor macrophages caused by reduction of a well-known pro-tumor macrophage promoter, CSF1R. Mechanistically, deletion of USP18 enhances interaction of the ubiquitin E3 enzyme NEDD4 and CSF1R and increases expression of an IFN-I-induced ubiquitin E2 enzyme, ubiquitin-conjugating enzyme H5 (UBCH5), which consequently enhances ubiquitination and degradation of CSF1R protein through the proteasome. Our results indicate the potential of targeting USP18 in macrophages as an IFN-based immunotherapy.

## RESULTS

### Generation of myeloid-lineage-specific USP18 conditional knockout mice

**Figure 1.**
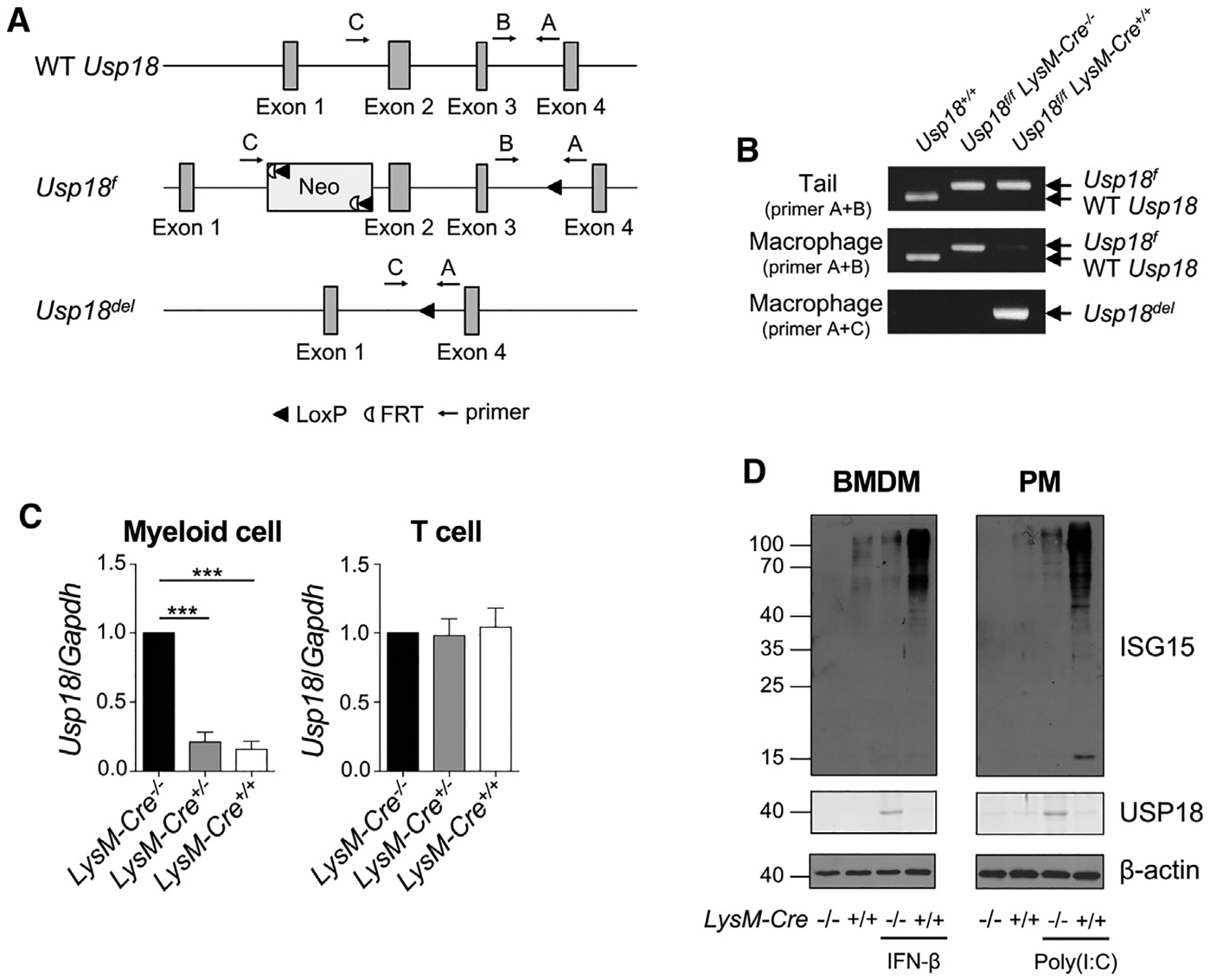
Deletion of Usp18 in myeloid-lineage cells (A) The wild-type *Usp18* locus (WT *Usp18*), the targeted *Usp18* locus in the *loxP*-flanked Usp18 allele (*Usp18*^*f*^), and the *Usp18* locus in myeloid-lineage cells from *Usp18*^*f/f*^ *LysM-Cre* mice (*Usp18*^*del*^). The positions of PCR primers A, B, and C are shown. The *loxP* sites flanked 4.4 kb. (B) PCR analysis of genomic DNA from tails (top) and peritoneal macrophages (middle and bottom) with the primers indicated in (A). Primers A and B were used in the top and middle images, and primers A and C were used in the bottom image. The PCR product obtained with primers A and B from the wild-type *Usp18* locus is 385 bp (WT *Usp18*) and that from the *Usp18*^*f*^ locus is 456 bp (*Usp18*^*f*^). In *Usp18*^*f/f*^ *LysM-Cre*^+/+^ mice, a band of 509 bp obtained with primers A and C indicates Cre-mediated deletion of *Usp18* (*Usp18*^*del*^). (C) Quantitative PCR analysis of mRNA from myeloid cells and T cells in *Usp18*^*f/f*^ *LysM-Cre*^−/−^, *Usp18*^*f/f*^ *LysM-Cre*^+/−^, and *Usp18*^*f/f*^ *LysM-Cre*^+/+^ mice. Myeloid cells from bone marrow and T cells from spleen were isolated. The values of *Usp18*^*f/f*^ *LysM-Cre*^−/−^ were considered as 1. Mean ± SEM, n = 3 in each group. One-way ANOVA multiple comparison test with *post hoc* Tukey test, ***p < 0.001. (D) Western blot of bone-marrow-derived macrophages (BMDM) with or without IFN-β treatment (100 U/mL, 24 h) and peritoneal macrophages (PM) from poly(I:C)-injected or uninjected mice (5 μg/g body weight, 48 h). Cells were harvested from *Usp18*^*f/f*^ *LysM-Cre*^−/−^ or *Usp18*^*f/f*^ *LysM-Cre*^+/+^ mice. Cell lysates were analyzed with the indicated antibodies. See also Figure S1.

To study USP18 in regulation of IFN signaling under different biological settings, we generated a conditional *Usp18* gene-KO mouse model (Figure 1A). USP18 is a potent inhibitor of IFN-I signal transduction and subsequent IFN-stimulated gene (ISG) expression.^19^ To investigate IFN-mediated changes in the TME to promote tumor growth, we generated myeloid-specific USP18-deficient mice by crossing conditional *Usp18*-KO mice (*Usp18*^*f/f*^) with *LysM-Cre* transgenic mice that express Cre recombinase in myeloid cells.^24^ Cre-mediated deletion was observed in the peritoneal macrophages (PMs) from *Usp18*^*f/f*^ *LysM-Cre*^+/+^ mice, but not in those from *Usp18*^+/+^ (wild type [WT]) or *Usp18*^*f/f*^ *LysM-Cre*^−/−^ mice (Figure 1B). No *Usp18* gene deletion was observed in the tail DNA from the *Usp18*^*f/f*^ *LysM-Cre*^+/+^ mice. Consistent with the results of genomic DNA, *Usp18* mRNA in CD11b^+^ myeloid cells from bone marrow was decreased in *Usp18*^*f/f*^ *LysM-Cre*^+/+^ mice, but not in CD3^+^ T cells (Figure 1C). As the downregulation of *Usp18* mRNA expression in *Usp18*^*f/f*^ *LysM-Cre*^+/−^ was comparable to that in *Usp18*^*f/f*^ *LysM-Cre*^+/+^ myeloid cells, we used both genotypes as myeloid-lineage-specific *Usp18*-KO mice (described as *Usp18*^*Δ/Δ*^ mice hereafter) in further experiments. Deletion of USP18 and enhanced IFN-I response as indicated by increased ISG15 expression were also confirmed at the protein level in both bone-marrow-derived macrophages (BMDMs) and PMs (Figure 1D). Hematological analyses showed that deletion of *Usp18* in myeloid cells did not affect normal hematopoiesis (Figure S1A). Furthermore, the deletion of *Usp18* did not affect the cell viability of myeloid cells (Figure S1B).

### Myeloid-lineage-specific deletion of USP18 suppresses tumor progression

**Figure 2.**
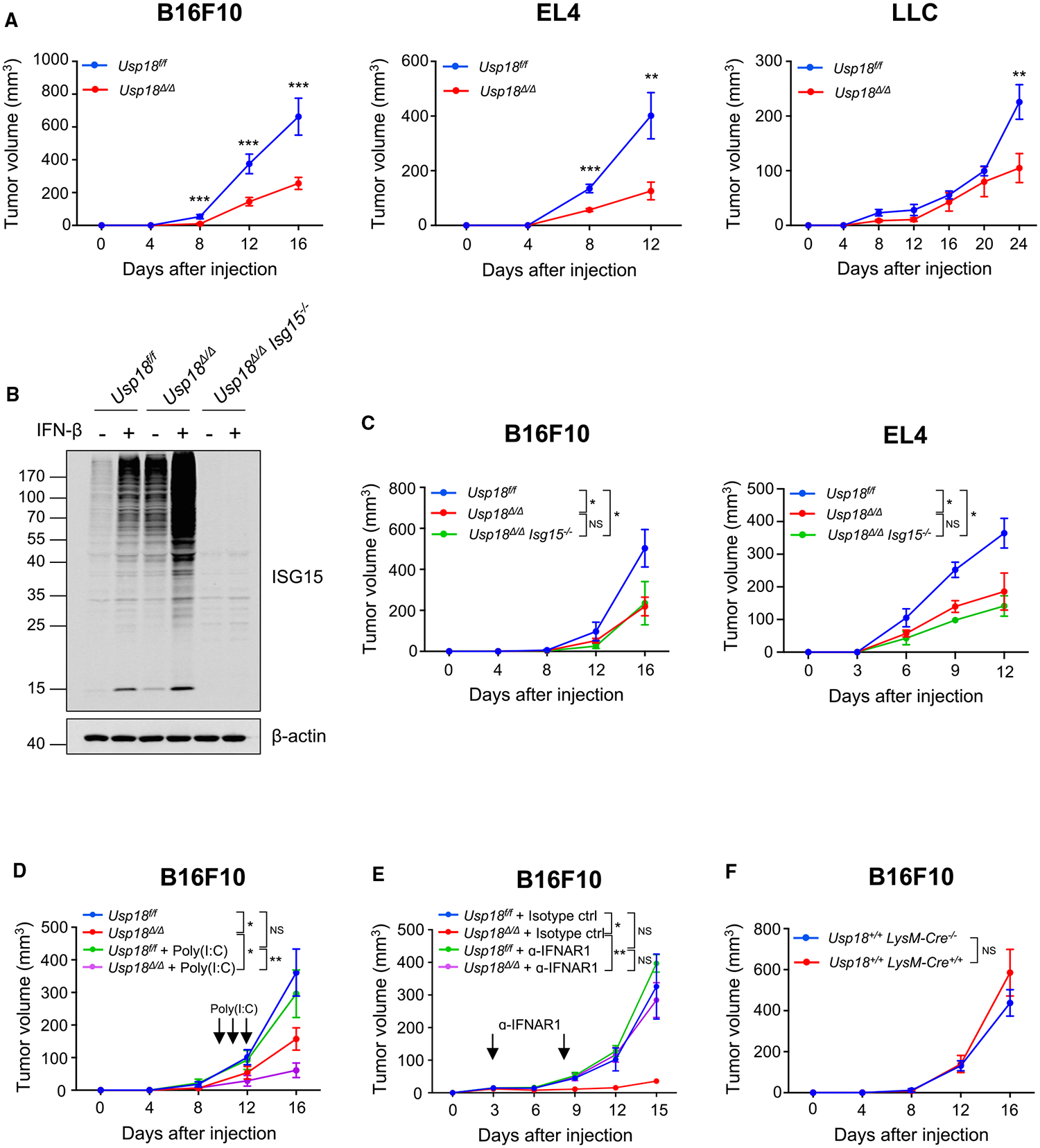
Suppressed tumor growth in *Usp18^Δ/Δ^* mice (A) B16F10 melanoma (1 × 10^5^), EL4 thymoma (5 × 10^6^), and LLC (5 × 10^5^) cells were subcutaneously injected into *Usp18*^*f/f*^ or *Usp18*^*Δ/Δ*^ mice (B16F10: *Usp18*^*f/f*^, n = 12; *Usp18*^*Δ/Δ*^, n = 17; EL4: *Usp18*^*f/f*^, n = 7; *Usp18*^*Δ/Δ*^, n = 9; LLC: *Usp18*^*f/f*^, n = 18; *Usp18*^*Δ/Δ*^, n = 10). (B) Western blot of bone-marrow-derived macrophages from *Usp18*^*f/f*^, *Usp18*^*Δ/Δ*^, and *Usp18*^*Δ/Δ*^ *Isg15*^−/−^ mice with or without IFN-β treatment (100 U/mL, 24 h). Cell lysates were analyzed with the indicated antibodies. (C) B16F10 melanoma (1 × 10^5^) or EL4 thymoma (5 × 10^6^) cells were subcutaneously injected into *Usp18*^*f/f*^, *Usp18*^*Δ/Δ*^, or *Usp18*^*Δ/Δ*^ *Isg15*^−/−^ mice (B16F10: *Usp18*^*f/f*^, n = 6; *Usp18*^*Δ/Δ*^, n = 8; *Usp18*^*Δ/Δ*^ *Isg15*^−/−^, n = 5; EL4: *Usp18*^*f/f*^, n = 7; *Usp18*^*Δ/Δ*^, n = 8; *Usp18*^*Δ/Δ*^ *Isg15*^−/−^, n = 4). (D) B16F10 melanoma cells (1 × 10^5^) were subcutaneously injected into *Usp18*^*f/f*^ or *Usp18*^*Δ/Δ*^ mice with or without poly(I:C) treatment. Poly(I:C) (5 μg/g body weight) was intraperitoneally injected on days 10, 11, and 12 (*Usp18*^*f/f*^, n = 5; *Usp18*^*Δ/Δ*^, n = 9; *Usp18*^*f/f*^ + poly(I:C), n = 5; *Usp18*^*Δ/Δ*^ + poly(I:C), n = 11). (E) B16F10 melanoma cells (1 × 10^5^) were subcutaneously injected into *Usp18*^*f/f*^ or *Usp18*^*Δ/Δ*^ mice treated with isotype control or anti-IFNAR1 antibodies. The antibodies (200 μg) were intravenously injected on days 3 and 8 (*Usp18*^*f/f*^ + isotype control, n = 6; *Usp18*^*Δ/Δ*^ + isotype control, n = 6; *Usp18*^*f/f*^ + α-IFNAR1, n = 6; *Usp18*^*Δ/Δ*^ + α-IFNAR1, n = 5). (F) B16F10 melanoma cells (1 × 10^5^) were subcutaneously injected into *Usp18*^+/+^ *LysM-Cre*^−/−^ or *Usp18*^+/+^ *LysM-Cre*^+/+^ mice (*Usp18*^+/+^ *LysM-Cre*^−/−^, n = 6; *Usp18*^+/+^ *LysM-Cre*^+/+^, n = 5). Mean ± SEM. Two-tailed unpaired t test and ordinary one-way ANOVA multiple comparison test with *post hoc* Tukey test were conducted for comparisons of two groups and more than two groups, respectively. NS, not significant; *p < 0.05, **p < 0.01, ***p < 0.001.

To elucidate the role of USP18 in myeloid cells on tumor progression, B16F10 melanoma, EL4 lymphoma, or LLC lung carcinoma cells were subcutaneously injected into *Usp18*^*f/f*^ and *Usp18*^*Δ/Δ*^ mice. Delayed tumor growth was observed in all of these tumor models in *Usp18*^*Δ/Δ*^ mice (Figure 2A). In addition to an inhibitory effect on IFN-I stimulation, USP18 deconjugates ubiquitin-like modifier ISG15 from ISGylated proteins.^14,17^ Therefore, we next explored whether USP18-regulated protein ISGylation in the TME contributed to this tumor-suppressive phenotype. We generated myeloid-specific *Usp18*-KO mice that have an additional deletion of *Isg15* (*Usp18*^*Δ/Δ*^ *Isg15*^−/−^) and repeated tumor growth studies using B16F10 and EL4 tumor cells. More ISG15-modified proteins were accumulated substantially in USP18-deficient myeloid cells (*Usp18*^*Δ/Δ*^) compared with control (*Usp18*^*f/f*^). In contrast, *Usp18*^*Δ/Δ*^ *Isg15*^−/−^ mice did not have protein ISGylation nor free ISG15 (Figure 2B). Compared with *Usp18*^*f/f*^ mice, tumor growth was significantly reduced in both *Usp18*^*Δ/Δ*^ mice and *Usp18*^*Δ/Δ*^ *Isg15*^−/−^ mice. We did not observe significant differences between *Usp18*^*Δ/Δ*^ mice and *Usp18*^*Δ/Δ*^ *Isg15*^−/−^ mice (Figure 2C). These results suggest that the function of USP18 in the regulation of protein ISGylation in myeloid cells is not involved in the enhanced anti-tumor activity observed in *Usp18*^*Δ/Δ*^ mice. To further analyze the function of USP18 in regulation of IFN response, we examined tumor growth with additional IFN stimulation by poly(I:C) treatment to stimulate endogenous IFN production. Poly(I:C) treatment did not affect tumor growth in *Usp18*^*f/f*^ mice; however, the anti-tumor effect was further enhanced by poly(I:C) treatment in *Usp18*^*Δ/Δ*^ mice (Figure 2D). Moreover, an IFN-I receptor (IFNAR1)-blocking antibody abrogated the anti-tumor effect observed in *Usp18*^*Δ/Δ*^ mice (Figure 2E). These results indicate that this anti-tumor effect is likely mediated by the enhanced IFN-I response and not by protein ISGylation in myeloid cells. In addition, we confirmed that there were no effects caused by Cre recombinase expression on tumor progression by comparing tumor growth between *Usp18*^+/+^ *LysM-Cre*^−/−^ mice and *Usp18*^+/+^ *LysM-Cre*^+/+^ mice (Figure 2F). Together, these results indicate that depletion of USP18 in myeloid cells enhances anti-tumor immune responses, which is not due to the increased protein modification by ISG15 and is likely related to the increased IFN-I response in the TME.

### Deletion of USP18 promotes macrophage polarization toward anti-tumor phenotypes

**Figure 3.**
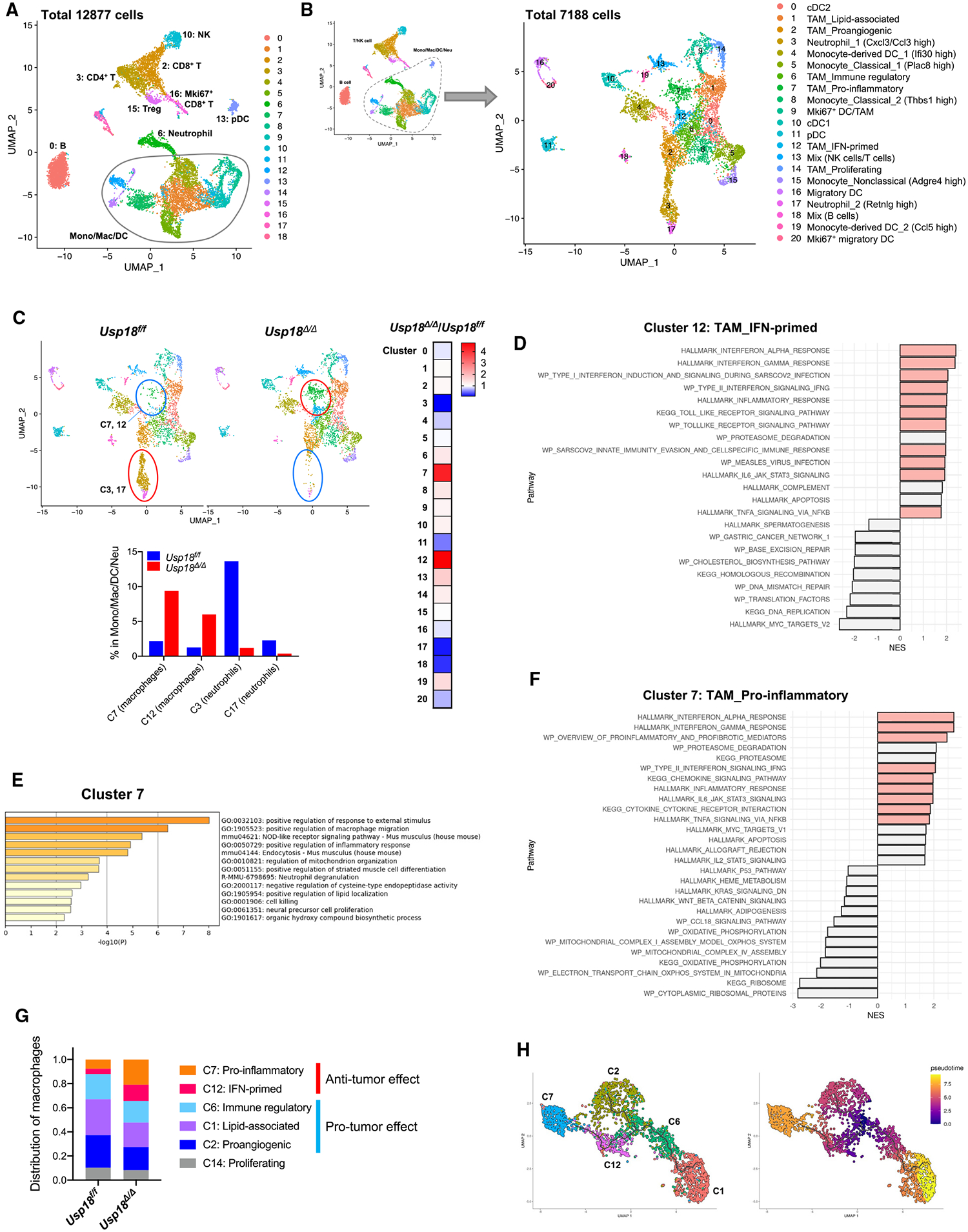
Increased anti-tumor macrophages in *Usp18^Δ/Δ^* mice B16F10 melanomas grown in *Usp18*^*f/f*^ and *Usp18*^*Δ/Δ*^ mice were harvested, and CD45^+^ cells isolated from the tumors were analyzed by single-cell RNA-seq. (A) UMAP plot of intratumoral CD45^+^ cells from the merged sample. (B) UMAP plot of the monocytes/macrophages/dendritic cells/neutrophils subpopulation (Mono/Mac/DC/Neu). (C) UMAP plot of the myeloid population split by *Usp18*^*f/f*^ and *Usp18*^*Δ/Δ*^ mice. Heatmap shows the changes in all the clusters. The bar graph shows the percentage of cells in the clusters with changes after the deletion of *Usp18*. (D) Pathway enrichment analysis of DEGs in cluster 12 by GSEA. The colored pathways indicate relevance to the annotated function of the cluster. (E) Pathway enrichment analysis of DEGs in cluster 7 by Metascape. (F) Pathway enrichment analysis of DEGs of *Usp18*^*Δ/Δ*^ in cluster 7. The colored pathways indicate relevance to the annotated function of the cluster. (G) Distribution of clusters in the macrophage population. (H) Trajectory inference analysis of TAM clusters (clusters 1, 2, 6, 7, and 12) analyzed by Monocle 3. See also Figures S2 and S3.

To examine the effects of myeloid-specific deletion of USP18 on the TME, single-cell RNA sequencing (RNA-seq) of tumor-infiltrating immune cells was performed. Tumor-infiltrating immune cells (CD45^+^ cells) from B16F10 tumors in *Usp18*^*f/f*^ and *Usp18*^*Δ/Δ*^ mice were sorted and analyzed. Both datasets were integrated, and non-linear dimensionality reduction was performed, shown in uniform manifold approximation and projection (UMAP) (Figure 3A). Each cluster was defined by using known markers^25–28^ (Figure S2A). Initial clustering analysis showed that there were fewer B cells (cluster 0) and neutrophils (cluster 6) in *Usp18*^*Δ/Δ*^ mice (Figure S2B). To increase resolution and define the myeloid-lineage populations more accurately, a subpopulation of monocytes, macrophages, dendritic cells, and neutrophils was further investigated by subclustering analysis (Figure 3B). A combination of automated and manual annotation methods was used for cell-type annotation. First, cells were annotated using SingleR^28^ with the ImmGen reference dataset.^29^ Then, if needed, the annotations were refined manually based on gene expression and TAM classification from a published article^30^ (Figures S2C and S2D). When *Usp18*^*Δ/Δ*^ mice were compared with *Usp18*^*f/f*^ mice, we observed an increase in clusters 7 and 12 (clusters of macrophages) and a decrease in clusters 3 and 17 (clusters of neutrophils) (Figure 3C). It is known that TAMs polarize and differentiate into functionally distinct subsets that change the TME. To further validate our cluster annotations and investigate how USP18-deletion-dependent alterations in macrophage clusters may correlate with TAM polarization and affect the TME, we performed pathway analysis on macrophage-annotated clusters using gene set enrichment analysis (GSEA).^31,32^ The top hit pathways in cluster 12 were IFN-α and IFN-γ responses (Figure 3D). Indeed, type I IFN-inducible genes were highly expressed in this cluster (Figure S3A). Accordingly, cluster 12 was annotated as IFN-primed TAMs with additional support from a recent review article on TAM classification.^30^ Similarly, other macrophage clusters were annotated based on enriched functional terms, highly expressed genes, and the recent TAM classification. Clusters 1, 2, 6, and 14 were annotated as lipid-associated TAMs, pro-angiogenic TAMs, immune-regulatory TAMs, and proliferating TAMs, respectively (Figure S3B). Interestingly, cluster 7 cells did not highly express a defined set of marker genes. Metascape analysis^33^ of reported cluster 7 markers reported several top hit pathways, including “positive regulation of inflammatory response” (Figure 3E); however, the functional associations were not as clear compared with the other TAM clusters. Therefore, we performed a subclustering analysis on cluster 7 for further characterization. The majority of the cells in cluster 7 were from *Usp18*^*Δ/Δ*^, and interestingly, cells from *Usp18*^*f/f*^ and *Usp18*^*Δ/Δ*^ conditions distributed into almost distinct clusters (Figure S3C). Pathway enrichment analysis on differentially expressed genes (DEGs) between *Usp18*^*f/f*^ and *Usp18*^*Δ/Δ*^ cells in cluster 7 revealed that the top hit pathways were pro-inflammatory responses, including IFN-α and IFN-γ responses (Figure 3F). Based on the results from Metascape and the subclustering analysis, cluster 7 was annotated as pro-inflammatory TAMs.

It has been reported that pro-inflammatory macrophages (cluster 7) and IFN-primed macrophages (cluster 12) have anti-tumor effects, whereas immune-regulatory macrophages (cluster 6), lipid-associated macrophages (cluster 1), and pro-angiogenic macrophages (cluster 2) have pro-tumor effects.^25,30,34–40^ The effect of proliferating macrophages (cluster 14) in mice is controversial, and further investigation is required. Thus, in the macrophage fraction of *Usp18*^*Δ/Δ*^ mice, we observe an increase in the macrophages with anti-tumor functions and a decrease in the macrophages with pro-tumor functions (Figure 3G).

To further analyze the polarization and differentiation of TAM subsets, we performed trajectory inference analysis using Monocle 3.^41–43^ TAMs frequently have immunosuppressive/pro-tumor phenotypes in the TME. Indeed, the higher infiltration/presence of TAMs in the tumor is associated with worse clinical outcomes in many cancer types.^3–5^ Using immune-regulatory TAMs (cluster 6) as a root, we identified three branching trajectories associated with distinct TAM clusters (Figure 3H). As the tumor progresses, immune-regulatory TAMs (cluster 6) differentiate into two types of pro-tumor TAMs along two branches: lipid-associated TAMs (cluster 1) and pro-angiogenic TAMs (cluster 2). Cells were also differentiated into anti-tumor TAMs along a third branch: IFN-primed TAMs (cluster 12) and pro-inflammatory TAMs (cluster 7). The clusters on this trajectory were increased by the deletion of *Usp18*, suggesting that the deletion of *Usp18* may induce the reprogramming of TAMs toward an anti-tumor phenotype. Taken together, single-cell RNA-seq analysis results revealed that USP18 deletion in myeloid cells enhances macrophage polarization toward anti-tumor/pro-inflammatory phenotypes, which likely contributes to the anti-tumor microenvironment.

### Deletion of USP18 downregulates CSF1R expression and promotes activation of CD8^+^ T cells

**Figure 4.**
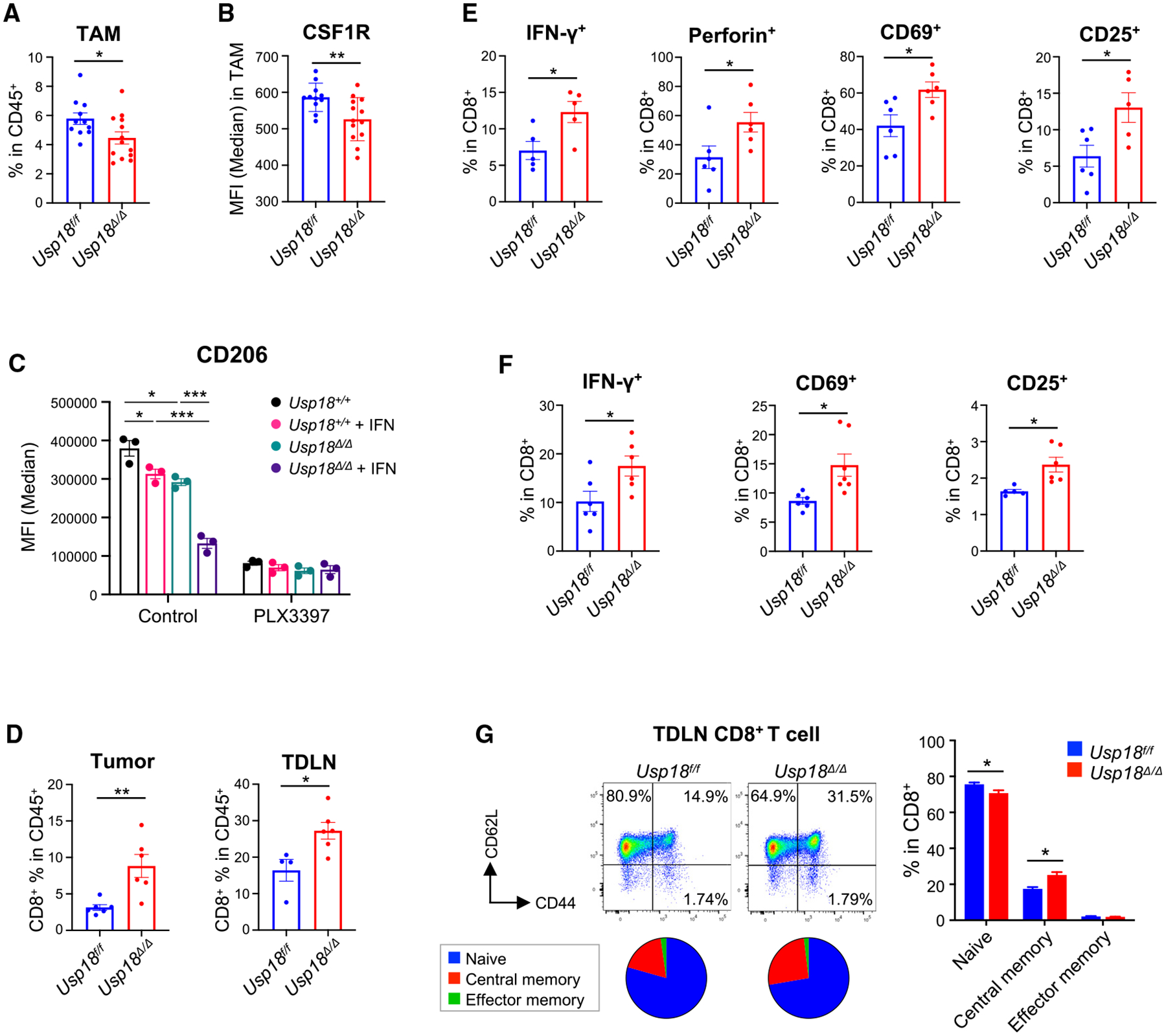
Decreased CSF1R expression in tumor-associated macrophages and increased activation of CD8^+^ T cells in *Usp18^Δ/Δ^* mice (A, B, and D‒G) B16F10 melanomas in *Usp18*^*f/f*^ and *Usp18*^*Δ/Δ*^ mice were harvested on day 16, and single-cell suspensions from tumors and tumor-draining lymph nodes (TDLNs) were analyzed by flow cytometry. (A and B) Percentage of TAMs in CD45^+^ cells (A) and MFI of CSF1R in TAMs (B) from tumor samples. *Usp18*^*f/f*^, n = 11; *Usp18*^*Δ/Δ*^, n = 13. Mean ± SEM. (C) BMDMs from *Usp18*^+/+^ or *Usp18*^*Δ/Δ*^ mice were treated with IFN-β (100 U/mL) for 24 h. As indicated, cells were treated with CSF1R inhibitor PLX3397 (50 μM) for 2 h prior to IFN-β treatment. BMDMs were also stimulated with IL-4 (40 ng/mL), IL-13 (20 ng/mL), and CSF1 (20 ng/mL) for 24 h to polarize toward an immunosuppressive phenotype and then analyzed by flow cytometry (n = 3). Mean ± SEM. (D‒F) Percentage of CD8^+^ T cells in CD45^+^ cells from tumor and TDLN samples (D), and cytokines and activation markers of CD8^+^ T cells from tumor (E) and TDLN (F) samples. *Usp18*^*f/f*^, n = 6; *Usp18*^*Δ/Δ*^, n = 6. Mean ± SEM. (G) Memory subsets of CD8^+^ T cells in TDLNs (naive, CD62L^+^CD44^−^; central memory, CD62L^+^CD44^+^; effector memory, CD62L^−^CD44^+^). *Usp18*^*f/f*^, n = 11; *Usp18*^*Δ/Δ*^, n = 13. Mean ± SEM. Two-tailed unpaired t test and ordinary one-way ANOVA multiple comparison test with *post hoc* Tukey test were conducted for comparisons of two groups and more than two groups, respectively. *p < 0.05, **p < 0.01, ***p < 0.001. See also Figure S4.

We next performed immune phenotyping in B16F10 tumor and tumor-draining lymph nodes (TDLNs) by flow cytometry (Figures S4A and S4B). Among myeloid subsets, TAMs (CD11b^+^F4/80^+^Gr-1^−^) and neutrophils (CD11b^+^Ly6G^+^) were decreased in *Usp18*^*Δ/Δ*^ mice (Figures 4A and S4C), which was consistent with the results from single-cell RNA-seq analysis (Figure 3C). We also observed that deletion of USP18 enhanced anti-tumor macrophage polarization in single-cell RNA-seq analysis (Figure 3G). One of the key molecules that regulates macrophage differentiation and polarization is colony-stimulating factor 1 (CSF1). CSF1 and other cytokines and chemokines recruit circulating monocytes and myeloid-derived suppressor cells (MDSCs) to tumor.^44^ Furthermore, CSF1 promotes macrophage survival and polarization signals that induce immunosuppressive macrophages.^45–48^ We, therefore, hypothesized that CSF1 signaling is involved in the phenotypes that we have observed and examined whether USP18 affects CSF1R expression by using flow cytometry. Compared with control (*Usp18*^*f/f*^), a significant reduction in CSF1R on TAMs was observed in *Usp18*^*Δ/Δ*^ mice (Figure 4B). Furthermore, to analyze the role of USP18 in CSF1R-mediated regulation of macrophage polarization, BMDMs from *Usp18*^+/+^ and *Usp18*^*Δ/Δ*^ mice were treated with the CSF1R inhibitor PLX3397 prior to IFN-I treatment. Then, macrophage polarization was analyzed by flow cytometry using CD206, a marker of pro-tumor/immunosuppressive macrophages.^49^ PLX3397 abrogated USP18 deficiency-induced reduction in pro-tumor/immunosuppressive macrophages (Figure 4C), supporting that USP18-mediated downregulation of CSF1R expression contributes to macrophage polarization.

In addition to the changes in TAM population, there were significant changes in T cell population. *Usp18*^*Δ/Δ*^ mice showed an increased frequency of CD8^+^ T cells in tumor and TDLN (Figure 4D). Furthermore, intracellular cytokines and lymphocyte activation markers were significantly increased in CD8^+^ T cells from the tumor (Figure 4E). Similar results were also observed in CD8^+^ T cells in TDLNs (Figure 4F). For induction of antigen-specific anti-tumor immune response, development of memory T cell subsets in lymph nodes is a crucial step. Interestingly, we observed enhanced memory CD8^+^ T cell formation in *Usp18*^*Δ/Δ*^ mice (Figure 4G). These results suggest that USP18 deletion leads to CSF1R downregulation in TAMs and a decrease in pro-tumor/immunosuppressive macrophages, contributing to enhanced anti-tumor macrophage polarization, consequently promoting a Th1-dominant TME and enhancing CD8^+^ T cell-mediated anti-tumor immunity, in agreement with previous reports.^6,50–52^

### IFN-I downregulates CSF1R protein expression, but not mRNA expression, in USP18-deficient macrophages

**Figure 5.**
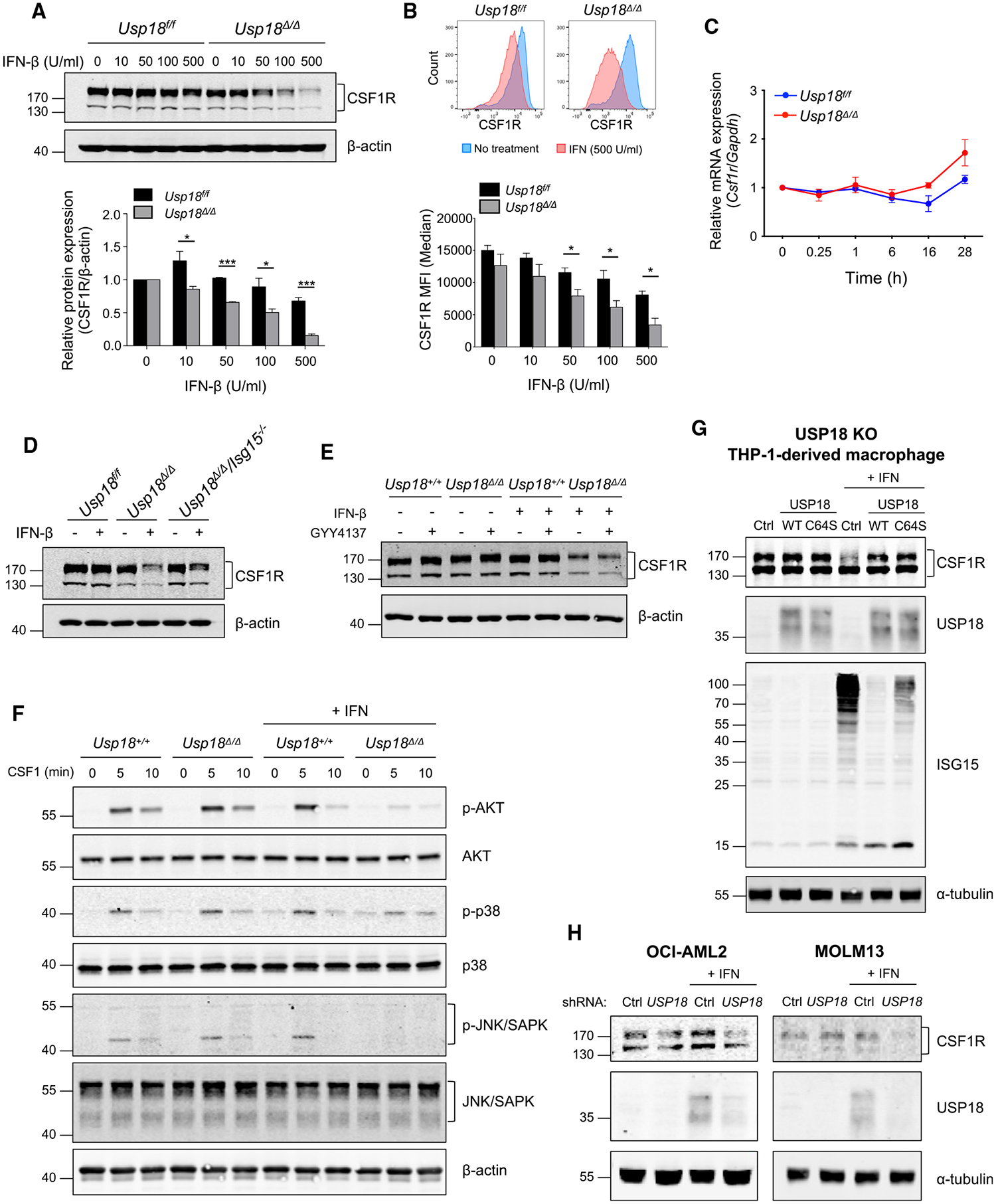
IFN-I-induced downregulation of CSF1R in a dose-dependent manner (A) Western blot of bone-marrow-derived macrophages (BMDMs) from *Usp18*^*f/f*^ and *Usp18*^*Δ/Δ*^ mice with or without IFN-β treatment for 24 h at the indicated concentrations. Cell lysates were analyzed with the indicated antibodies (n = 3). Mean ± SEM, two-tailed unpaired t test, *p < 0.05, ***p < 0.001. (B) CSF1R cell-surface expression on BMDMs from *Usp18*^*f/f*^ and *Usp18*^*Δ/Δ*^ mice with or without IFN-β treatment for 24 h at the indicated concentrations was analyzed by flow cytometry (n = 3). Mean ± SEM, two-tailed unpaired t test, *p < 0.05. (C) *Csf1r* mRNA expression in BMDMs from *Usp18*^*f/f*^ and *Usp18*^*Δ/Δ*^ mice with or without IFN-β treatment (100 U/mL for the indicated times) was analyzed by RT-qPCR (n = 3). (D) Western blot of BMDMs from *Usp18*^*f/f*^, *Usp18*^*Δ/Δ*^, and *Usp18*^*Δ/Δ*^ *Isg15*^−/−^ mice with or without IFN-β treatment (100 U/mL for 24 h). (E) BMDMs from *Usp18*^*f/f*^ *UBC-Cre-ERT2* mice were treated with β-estradiol (2 μM) for 48 h. Cells were then treated with IFN-β (100 U/mL) for 24 h with or without NF-κB inhibitor GYY4137 (100 μM) for 2 h prior to IFN-β treatment. Cell lysates were analyzed with the indicated antibodies by western blotting. (F) BMDMs from *Usp18*^*f/f*^ and *Usp18*^*Δ/Δ*^ mice were treated with IFN-β (100 U/mL for 24 h) and with CSF1 (50 ng/mL) for the indicated times after CSF1 starvation for 4 h. Cell lysates were analyzed with the indicated antibodies by western blotting. (G) USP18-KO THP-1-derived macrophages with the expression of empty vector control, wild-type USP18, and USP18 C64S were treated with IFN-I (1,000 U/mL for 24 h) and analyzed by western blotting. (H) CSF1R in OCL-AML2 and MOLM13 cells infected with control or *USP18* short hairpin RNA (shRNA) with or without IFN-I (1,000 U/mL for 24 h) was analyzed by western blotting.

To elucidate further details of the downregulation of CSF1R in USP18-deficient macrophages, we investigated the effects of IFN-I on CSF1R expression *in vitro*. BMDMs from *Usp18*^*f/f*^ or *Usp18*^*Δ/Δ*^ mice were utilized for the analyses. The dose-dependent IFN-I-induced downregulation of total CSF1R protein expression was detected by western blotting (Figure 5A). Flow cytometric analysis showed that cell-surface expression of CSF1R was also downregulated (Figure 5B). These results support the observation in the TME (Figure 4B). Although downregulation of cell-surface CSF1R expression was also observed in BMDMs from *Usp18*^*f/f*^ mice upon prolonged IFN treatment, the decrease was much greater in BMDMs from *Usp18*^*Δ/Δ*^ mice (Figure 5B). To check whether USP18 regulates CSF1R at the transcript level, we performed RT-qPCR analysis. There was no significant decrease in *Csf1r* mRNA (Figure 5C), indicating that the downregulation of CSF1R happened mainly at the post-transcriptional level. Consistent with this, there was no difference in *Csf1r* expression in single-cell RNA-seq analysis (Figure S2E). Furthermore, USP18 and ISG15 double-KO BMDMs also showed CSF1R downregulation to the same degree (Figure 5D), supporting the idea that USP18-mediated regulation of protein ISGylation is not involved in the reduction of CSF1R protein.

We previously reported that the deletion of USP18 activates the expression of NF-κB-regulated genes.^19^ As NF-κB is one of the major transcription factors regulating the expression of pro-inflammatory genes, we investigated whether the polarization toward the pro-inflammatory phenotype observed in *Usp18*^*Δ*/*Δ*^ macrophages caused by CSF1R downregulation relies on the NF-κB pathway. The NF-κB inhibitor GYY4137 did not affect CSF1R downregulation in *Usp18*^*Δ/Δ*^ BMDMs (Figure 5E), suggesting that NF-κB pathways were not involved in the IFN-mediated downregulation of CSF1R. The downregulation of CSF1R expression also led to the suppression of PI3K and MAPK signaling, the major downstream pathways of CSF1R, in *Usp18*^*Δ/Δ*^ macrophages (Figure 5F). To investigate if IFN-I-induced downregulation of CSF1R can also be observed in a human system, USP18-KO THP-1-derived macrophages were analyzed. CSF1R expression was downregulated after IFN-I treatment in USP18-KO THP-1 (Figure 5G). As USP18 also functions as a deconjugating enzyme, it may mediate the deubiquitination of CSF1R, which inhibits its protein degradation. To examine if the deconjugating activity of USP18 is involved in the regulation of CSF1R expression, either WT USP18 or an enzymatically inactive USP18 C64S mutant was expressed in the USP18-KO THP-1 cells. The CSF1R protein level was restored with the reexpression of either WT USP18 or USP18 C64S in the USP18-KO THP-1 cells, supporting the idea that the enzymatic activity of USP18 is not responsible for the downregulation of CSF1R (Figure 5G). We also confirmed that the IFN-I-mediated downregulation of CSF1R was observed in other human myeloid cell lines, OCI-AML2 and MOLM13 (Figure 5H). Together, these data suggest that the deletion of USP18 mediates the downregulation of CSF1R at the post-transcriptional level through enhanced IFN-I response.

### NEDD4 interacts with USP18 and mediates ubiquitin-dependent proteasomal degradation of CSF1R

**Figure 6.**
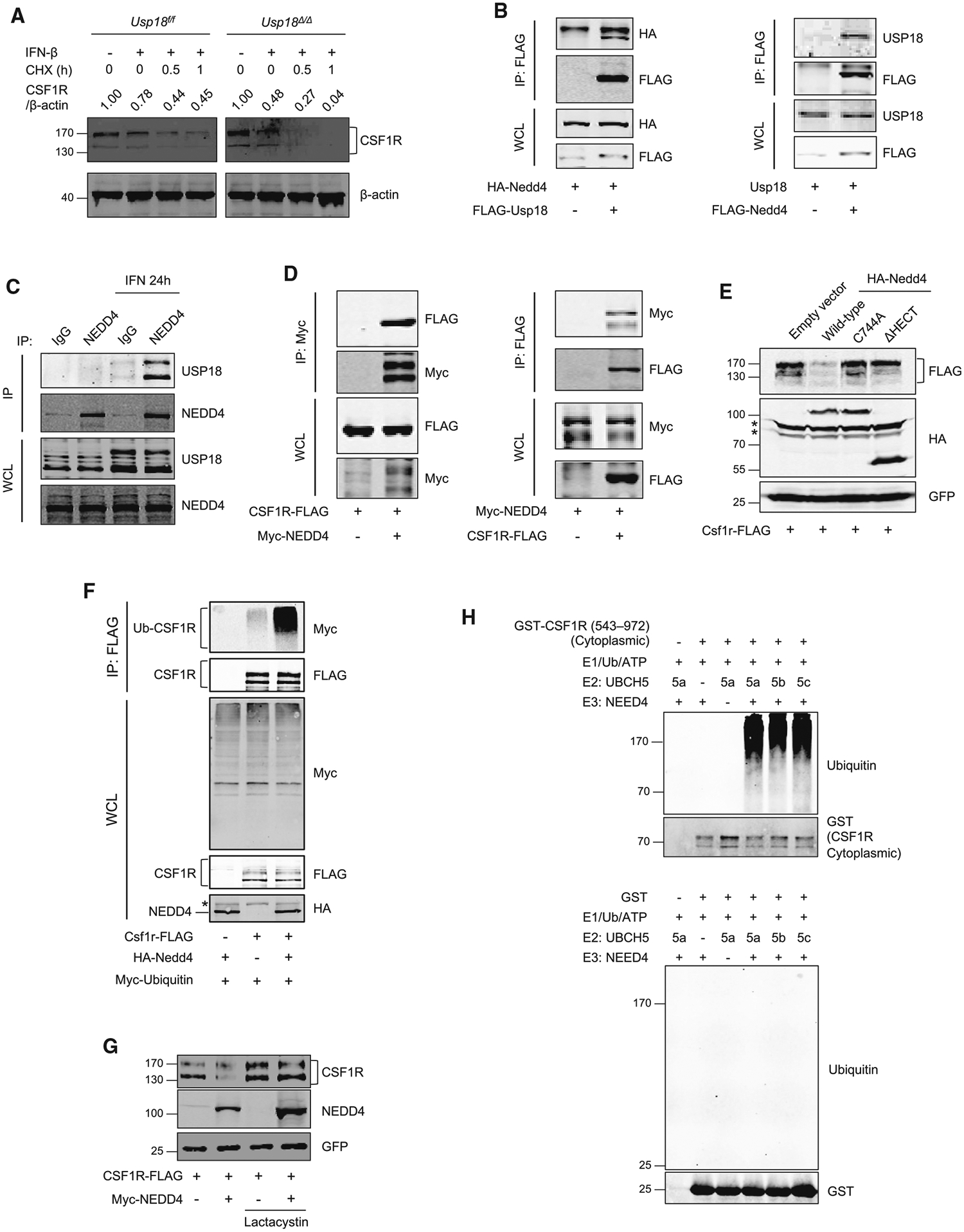
NEDD4-mediated ubiquitin-dependent proteasomal degradation of CSF1R (A) Bone-marrow-derived macrophages (BMDMs) from *Usp18*^*f/f*^ and *Usp18*^*Δ/Δ*^ mice and wild type were treated with CHX (50 μg/mL) for the indicated times, and CSF1R expression was analyzed by western blotting. (B) HEK293T cells were transfected for 24 h as indicated. Protein interaction was analyzed by co-immunoprecipitation. (C) Interaction of endogenous USP18 and NEDD4 in THP-1-derived macrophages with or without IFN-I (1,000 U/mL for 24 h) was analyzed by co-immunoprecipitation by using anti-NEDD4 antibody. (D) HEK293T cells were transfected as indicated. Twenty-four hours after transfection, protein interaction was analyzed by co-immunoprecipitation. (E) HEK293T cells were transfected with FLAG-CSF1R and either wile-type or mutated HA-NEDD4 for 36 h. GFP was transfected as a control. Asterisks (*) indicate non-specific signals. (F) HEK293T cells were transfected as indicated with lactacystin for 24 h. Ubiquitination of mouse CSF1R was analyzed after immunoprecipitation. Asterisk (*) indicates a non-specific signal. (G) HEK293T cells were transfected as indicated with or without lactacystin for 24 h. Human CSF1R expression was analyzed by western blotting. GFP was transfected as a control. (H) *In vitro* ubiquitination assay of CSF1R substrate protein with E2 enzymes UBCH5a/5b/5c and E3 enzyme NEDD4. Reaction mixtures as indicated were incubated at 30°C for 1 h. The eluate was analyzed by western blotting.

To clarify further mechanisms of downregulation of CSF1R, a protein stability cycloheximide (CHX) chase assay was performed. It revealed that degradation of CSF1R was faster in BMDMs from *Usp18*^*Δ/Δ*^ mice than in control cells, indicating that USP18 deletion enhanced the process of CSF1R degradation (Figure 6A). Protein ubiquitination plays a major role in degradation of cellular proteins. One ubiquitin E3 ligase, neural precursor cell-expressed, developmentally downregulated 4 (NEDD4), was previously detected as a USP18-interacting protein in our yeast two-hybrid study. NEDD4 is widely expressed in mammalian tissues, and more than 50 proteins are reported as targets of NEDD4, including IGF-1R, PTEN, and EGFR.^53^ We hypothesized that NEDD4 also mediates CSF1R degradation and that USP18 modulates the process. We first used co-immunoprecipitation to analyze the interaction of USP18 and NEDD4. The interaction between exogenously expressed USP18 and NEDD4 was observed (Figure 6B). In addition, we detected the interaction between endogenous USP18 and NEDD4 in THP-1-derived macrophages (Figure 6C). Furthermore, our reciprocal co-immunoprecipitation results demonstrate the interaction of NEDD4 and CSF1R (Figure 6D), suggesting that CSF1R is a ubiquitination substrate of NEDD4.

To examine whether NEDD4 E3 ligase mediates degradation of CSF1R, WT and mutant NEDD4 without enzyme activity were expressed with CSF1R. Since NEDD4 is an E3 in the HECT-domain ubiquitin ligase family, we generated the HECT-domain-deletion (ΔHECT) mutant and C744A-enzymatic-active-site mutant. CSF1R was downregulated by only WT NEDD4 and not the two mutants of NEDD4 (Figure 6E). A proteasome inhibitor, lactacystin, was used to further examine the involvement of the ubiquitin-proteasome pathway in the NEDD4-mediated degradation of CSF1R. Enhanced ubiquitination of CSF1R (Figure 6F) and restoration of CSF1R protein level (Figure 6G) were observed with lactacystin treatment. Furthermore, *in vitro* ubiquitination assays showed that CSF1R but not GST protein was able to be ubiquitinated by E3 ligase NEDD4 (Figure 6H). These data demonstrate that NEDD4 mediates the degradation of CSF1R via the ubiquitin-proteasome pathway.

### USP18 impairs degradation of CSF1R by inhibiting CSF1R-NEDD4 interaction and regulating ubiquitin E2 UBCH5 expression

**Figure 7.**
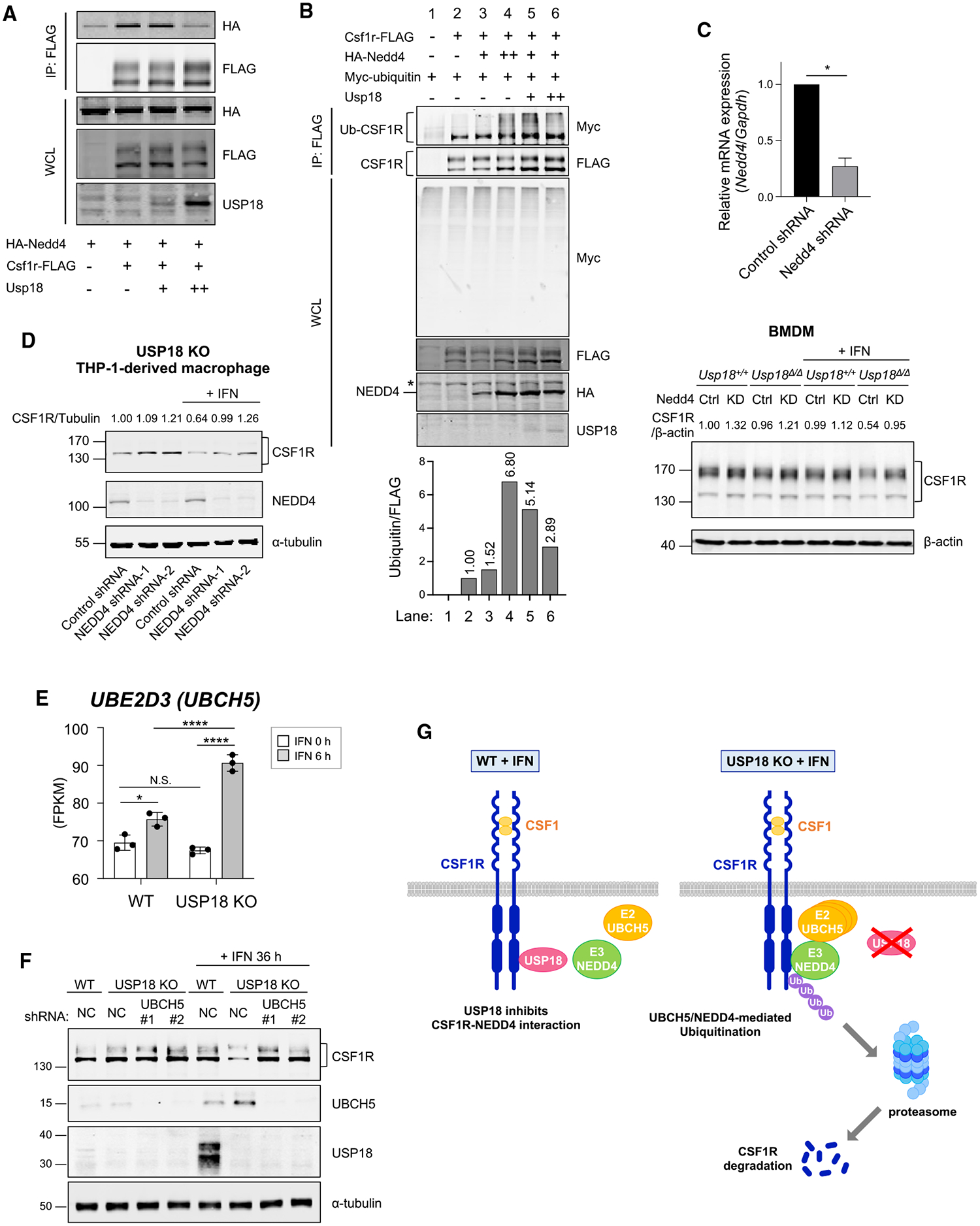
Inhibitory effect of USP18 on NEDD4-mediated ubiquitination of CSF1R (A) Interaction of CSF1R and NEDD4 in the presence of USP18 was analyzed. HEK293T cells were transfected as indicated for 24 h. Co-immunoprecipitation followed by western blotting was performed. (B) HEK293T cells were transfected as indicated for 24 h. All samples were treated with lactacystin (10 μM) for 2 h. Co-immunoprecipitation followed by western blotting was performed. Asterisk (*) indicates a non-specific signal. (C) Nedd4-knockdown or control bone-marrow-derived macrophages (BMDMs) from *Usp18*^*f/f*^ *UBC-Cre-ERT2* mice were treated with β-estradiol (2 μM) for 48 h. Knockdown of Nedd4 was confirmed by qPCR (top). CSF1R expression in the BMDMs with or without IFN-β treatment (100 U/mL for 24 h) was analyzed by western blotting (bottom). Mean ± SEM, two-tailed unpaired t test, *p < 0.05. (D) NEDD4 was knocked down by shRNA in USP18-knockout THP-1-derived macrophages. The expression of CSF1R was analyzed by western blotting. (E) *UBE2D3* expression in WT or USP18-KO THP-1 cells with or without IFN-I treatment analyzed by bulk RNA-seq (n = 3). Mean ± SD. One-way ANOVA multiple comparison test with *post hoc* Tukey test; N.S., not significant; *p < 0.05, ****p < 0.0001. (F) UBCH5C and CSF1R expression in THP-1-derived macrophages with knockdown of UBCH5C was analyzed by western blotting. (G) Scheme of the inhibitory effect of USP18 on NEDD4-dependent downregulation of CSF1R.

To reveal how USP18 modulates the NEDD4-mediated degradation of CSF1R, the interaction of CSF1R and NEDD4 was analyzed in the presence of USP18. USP18 inhibited the interaction of CSF1R and NEDD4 in a dose-dependent manner (Figure 7A). We then analyzed USP18 in NEDD4-mediated ubiquitination in the presence of the proteasome inhibitor lactacystin. NEDD4-mediated ubiquitination of CSF1R (Figure 7B, lanes 3 and 4) was impaired in the presence of USP18 (Figure 7B, lanes 5 and 6). These data indicate that USP18 inhibits the interaction of CSF1R and NEDD4, which diminishes the NEDD4-mediated ubiquitination of CSF1R and results in the inhibition of CSF1R degradation. To confirm these findings, NEDD4 was knocked down in BMDMs and THP-1-derived macrophages. Knockdown of NEDD4 showed restoration of CSF1R expression in USP18-KO (*Usp18*^*Δ/Δ*^) BMDMs (Figure 7C). Also, IFN-induced downregulation of CSF1R in USP18-KO cells was restored by the knockdown of NEDD4 (Figure 7D). Together, these results support the idea that NEDD4 is a modulator of CSF1R downregulation. Interestingly, although NEDD4-mediated CSF1R degradation was enhanced by IFN-I treatment, NEDD4 itself is not induced by IFN-I, suggesting that other regulators in the ubiquitin-proteasome pathway may be responsible for IFN-I-mediated CSF1R degradation. We analyzed an IFN-I-inducible E2 ubiquitin-conjugating enzyme, UBCH5.^54^ Our RNA-seq analysis of THP-1 cells showed that the expression of *UBE2D3* (UBCH5) was significantly upregulated in parental and USP18-KO cells upon IFN-I treatment (Figure 7E). Furthermore, UBCH5 protein was induced and CSF1R was downregulated in USP18-KO THP-1-derived macrophages after IFN-I treatment (Figure 7F). Importantly, the downregulation of CSF1R was restored by knocking down UBCH5 expression. Together, these data show that UBCH5 expression is enhanced upon IFN-I stimulation and that UBCH5 mediates IFN-I-dependent degradation of CSF1R.

Figure 7G is the scheme of the mechanism we propose based on the current studies. IFN-Is are produced in the TME, which induces the expression of USP18 and the ubiquitin E2 enzyme UBCH5. In WT macrophages, USP18 inhibits the interaction of CSF1R and the ubiquitin E3 NEDD4, which suppresses ubiquitination and subsequent degradation of CSF1R. On the other hand, in USP18-KO macrophages, NEDD4 binds to CSF1R, which enhances UBCH5 and NEDD4-mediated ubiquitination and subsequent degradation of CSF1R, leading to inhibition of polarization toward immunosuppressive macrophages.

## DISCUSSION

In this study, we sought to reveal the functions of IFN-I in myeloid cells during tumor development. We utilized a myeloid-lineage-specific USP18-deletion mouse model, which showed that deletion of USP18 delayed tumor growth. Our single-cell transcriptional characterization of tumor-infiltrated immune cells uncovered differences after USP18 depletion, including an increase in anti-tumor macrophages. CSF1R expression, a key signal for the polarization toward an immunosuppressive/pro-tumorigenic phenotype, in macrophages was downregulated by USP18 KO *in vivo* and *in vitro*. We found that NEDD4 is a ubiquitin E3 ligase for CSF1R, and USP18 inhibits its degradation by interfering with the interaction of CSF1R and NEDD4. Furthermore, depletion of the negative regulator of IFN-I signaling, USP18, enhanced expression of ubiquitin E2 UBCH5. Together, our data demonstrate that suppression of USP18 promotes UBCH5 and NEDD4-mediated proteasome degradation of CSF1R, leading to an increase in anti-tumor macrophages in the TME. This is, at least in part, a mechanism of enhanced anti-tumor activity observed in USP18-KO mice.

A previous report demonstrated that deletion of adenosine deaminase acting on RNA 1 (ADAR1), a suppressor of IFN-I response, decreased immunosuppressive macrophages and myeloid-derived suppressor cells and enhanced anti-tumor immunity, which indicates the importance of IFN-I in TME.^26^ Also, it was reported that IFN-Is inhibited the generation of TAMs by using an IFN-α/β receptor-KO mouse.^55^ These authors also showed that IFN-Is significantly inhibited the generation of bone-marrow-cell-derived macrophages in response to CSF1 *in vitro*. This may be explained by our finding that IFN-I mediated downregulation of CSF1R (Figure 5). We previously reported that deletion of USP18 activates expression of NF-κB-regulated genes.^19^ In this study, we observed that IFN-I-mediated CSF1R downregulation in USP18-KO macrophages was not regulated by NF-κB (Figure 5E). However, NF-κB is known to be one of the key transcription factors related to polarization toward anti-tumor/pro-inflammatory macrophages.^56^ NF-κB regulates the expression of a large number of inflammatory genes, including TNF-α, IL-1β, cyclooxygenase-2 (COX-2), IL-6, and IL-12p40.^57^ Therefore, it is likely that anti-tumor/pro-inflammatory macrophage polarization observed in USP18-KO macrophages also relies on the NF-κB pathway in addition to the CSF1R-mediated regulation.

TAMs are good targets for cancer therapy. In addition to their immunosuppressive functions in the TME, TAMs are also known to mediate resistance to standard therapies, including chemo-therapy and radiation therapy.^48,58–60^ As CSF1 is required for macrophage differentiation, different types of CSF1R blockades have been approved for clinical use, including monoclonal antibodies against CSF1R and tyrosine kinase inhibitors. In our current study, deletion of USP18 in macrophages downregulated CSF1R expression on TAMs and reduced the frequency of immunosuppressive TAMs in the TME. More importantly, we demonstrated that deletion of USP18 created an anti-tumor microenvironment by repolarization of TAMs toward anti-tumor macrophages (Figures 3 and 4). The above therapeutic applications support the clinical impact of our current studies. Accumulating evidence suggests the importance of macrophage polarization in tumor progression. For example, Pyonteck et al. reported that CSF1R blockade reduces immunosuppressive macrophage polarization, which improved disease outcomes in their glioma models.^45^ Other reports also demonstrated the impact of TAM polarization on tumor progression,^61^ supporting the idea that reprogramming of TAMs toward the anti-tumor phenotype induced by USP18 deletion has potential for clinical use.

Tong et al. reported that IFN-I downregulates CSF1R expression via miR-155.^62^ IFN-I inhibits differentiation of Ly6C^+^ monocytes to TAMs by inhibiting upregulation of CSF1R in monocytes during differentiation to macrophages. Consistent with our finding shown in Figure 5C, mRNA expression of *Csf1r* was not reduced after IFN treatment in the report. They found that the inhibitory effect of IFN on CSF1R occurs at the level of mRNA translation mediated by IFN-induced miR-155. In our current study, we revealed another mechanism of CSF1R regulation mediated by USP18 and NEDD4.

Reduction of CSF1R on cell membranes has been reported by shedding with TNF-α-converting enzyme TACE^63^ and γ-secretase^64^ and by CSF1 or Toll-like receptor (TLR) agonist-stimulated internalization and lysozyme degradation.^65,66^ However, such changes happened within 30 min after stimulation. In contrast, IFN-I-dependent downregulation of CSF1R takes much longer, supporting the idea that IFN-I induces a mechanism of degradation different from the one induced by CSF1 or TLR agonists. Also, proto-oncoprotein c-Cbl is reported as an E3 ligase for CSF1R.^67–69^ This c-Cbl-induced ubiquitination of CSF1R leads to internalization and endocytosis of the receptor, followed by receptor degradation in lysosomes.^70^ Therefore, this degradation process is completely separate from our findings. Here, we identified NEDD4 as a ubiquitin E3 ligase for CSF1R and demonstrated that UBCH5 served as its E2 enzyme. The UBCH5 gene is a known IFN-I-inducible gene, whereas the NEDD4 gene is not.^54^ Therefore, under the circumstances of enhanced IFN-I signaling caused by USP18 deletion, IFN-I-inducible E2 enzyme UBCH5 likely enhances the activity of E3 ligase NEDD4, leading to degradation of CSF1R via the ubiquitin-proteasome system.

Since NEDD4 is frequently overexpressed in cancers, including prostate, bladder, and colon cancers,^71^ NEDD4 was originally thought to be an oncogene. However, recently, NEDD4 is thought to have dual roles as an oncogene and a tumor suppressor in cancers by mediating the ubiquitination of substrates that have a variety of functions.^72^ Furthermore, accumulating evidence suggests that NEDD4 also has important roles in the immune system. NEDD4 enhances T cell activation and proliferation by promoting ubiquitin-mediated degradation of Cbl-b, which negatively regulates T cell activation.^73–75^ With regard to B cells, NEDD4 promotes the activation of the CD40-Akt pathway by ubiquitination of TRAF3, inducing immunoglobulin class switching, which is essential for humoral immunity.^76^ It has also been reported that NEDD4 plays an important role in macrophages during the innate immune response and inflammation. NEDD4 regulates TNF-α expression from macrophages by mediating ubiquitination of p38α^77^ and enhances killing of intracellular bacterial pathogens by promoting autophagy.^78^ Furthermore, Nuro-Gyina et al. showed that NEDD4 is essential for anti-fungal innate immune response by using *Nedd4*^*f/f*^ *LysM-Cre* mice.^79^ However, our current study shows the function of NEDD4 in TAMs.

Given that USP18 inhibits NEDD4-mediated ubiquitination and subsequent degradation of CSF1R, we hypothesized that USP18 may also regulate NEDD4-mediated ubiquitination of other target proteins. It has been reported that RAP2A is one of the targets of the NEDD4-mediated ubiquitination.^80^ To investigate if the interaction of USP18 and NEDD4 affects the ubiquitin conjugation to RAP2A, NEDD4, RAP2A, ubiquitin, and USP18 were co-expressed in HEK293T cells. We confirmed that NEDD4 enhanced ubiquitination of RAP2A; however, it was not affected in the presence of USP18, suggesting that not all of the NEDD4-mediated ubiquitination was inhibited by USP18. This could be due to the difference in other ubiquitination components, such as E2 enzymes. RAP2A is just one example among more than 20 of the reported substrates of NEDD4, and there may be other targets of NEDD4-mediated ubiquitination that are regulated by USP18. If so, USP18 could be involved in other diseases and be a potential therapeutic target to enhance the function of NEDD4. Further investigation is warranted.

Our findings suggest that targeting USP18 in macrophages has the potential to reprogram TAM to enhance anti-tumor activity in different types of cancers. This would be a good therapeutic strategy for the following reasons. In most tumors, myeloid cells are the most abundant cell types among tumor-infiltrating immune cells. Although T cell-based anti-tumor therapies, such as checkpoint blockade immunotherapy, are currently used, one of the limitations of T cell-targeting therapy is the low number of tumor-infiltrating T cells. As myeloid cells usually account for 30%–50% of infiltrating immune cells in tumors, it would be feasible and more efficient to target them to regulate T cells as presented in our current studies. In addition, circulating monocytes are one of the main sources of macrophages in tumors,^81^ which can be targeted by USP18 depletion as well. Importantly, deletion of USP18 in myeloid-lineage cells did not show any negative effects on normal hematopoiesis (Figure S1). Given these advantages, targeting USP18 in myeloid cells is potentially a promising therapeutic strategy across different types of cancers and warrants further investigation.

### Limitations of the study

In the current study, the tumor-infiltrating immune cells were analyzed only by single-cell RNA-seq. There was no information on protein expression, which requires investigation by cellular indexing of transcriptomes and epitopes by sequencing (CITE-seq) or other, similar methods to support our conclusion. Also, cellular neighborhoods in the TME remain to be investigated by digital-spatial analysis. Moreover, additional functional analysis of the different macrophage populations will further improve the annotation of the macrophage clusters and help us to understand the more precise mechanism of USP18-mediated reprogramming of macrophages.

## STAR★METHODS

### RESOURCE AVAILABILITY

#### Lead contact

Further information and requests for resources and reagents should be directed to and will be fulfilled by the lead contact, Dong-Er Zhang (d7zhang@health.ucsd.edu).

#### Materials availability

The following plasmids were generated in this study: Plasmid: pCX4-bsr-mouse Usp18, pCX4-bsr -FLAG-mouse Usp18, pCX4-bsr-human USP18 (sgRNA resistant), pCX4-bsr-human USP18 C64S (sgRNA resistant), pCAG-mouse Csf1r-FLAG, pCAG-human CSF1R-FLAG, pcDNA-Myc-human NEDD4, pcDNA-HA-mouse Nedd4, pcDNA-HA-mouse Nedd4 (C744A mutant), pcDNA-HA-mouse Nedd4 (HECT domain deletion mutant), pcDNA-FLAG-mouse Nedd4, pcDNA-Myc-Ubiquitin, pSUPER.retro.puro-human USP18 shRNA, pLKO.1 Negative control (GFP).

All materials generated in this study are available from the lead contact with a completed materials transfer agreement.

#### Data and code availability

- Single-cell and bulk RNAseq data have been deposited at GEO and are publicly available as of the date of publication. Accession numbers are listed in the key resources table.
- This paper does not report original code.
- Any additional information required to reanalyze the data reported in this paper is available from the lead contact upon request.

### EXPERIMENTAL MODELS AND STUDY PARTICIPANT DETAILS

#### Mouse studies

All animal studies were approved by the Institutional Animal Care and Use Committee of the University of California, San Diego (S07271). All mice were housed and bred in a specific pathogen-free vivarium at UCSD Moores Cancer Center accredited by the American Association for the Accreditation of Laboratory Animal Care. *Usp18*^*f/f*^ mice were generated by Ingenious Targeting Laboratory as previously described.^19^ *LysM-Cre* and *UBC-Cre-ERT2* mice were purchased from The Jackson Laboratory. *Isg15* knockout mice were from Dr. Klaus-Peter Knobeloch.^82^ Six to ten-week-old female and male mice were used for experiments. Littermates of the same sex were randomly assigned to experimental groups. Hematological parameters in peripheral blood were analyzed by using Vet abc Plus (scil animal care company).

For the establishment of tumor models, 1 × 10^5^ B16F10 melanoma, 5 × 10^6^ EL4 lymphoma, or 5 × 10^5^ LLC lung carcinoma were subcutaneously injected into the right flank. Tumor diameter was measured every 3–4 days with an electronic caliper and reported as a volume using the formula; tumor volume = (length × width × height)/2. For Poly(I:C) treatment, 5 μg/g body weight of Polyinosinic-polycytidylic acid sodium salt (Sigma-Aldrich) was intraperitoneally injected starting on Day 10 daily for 3 days. For IFNAR1 blocking experiment, α-IFNAR1 or isotype control antibodies (Bio X cell) were intravenously injected on Day 3 and 8 (200 μg/dose).

#### Cell lines and primary cell culture

B16F10 from Dr. Michiko Fukuda (Sanford Burnham Prebys Institute), EL4 from American Type Culture Collection (ATCC), and Lewis Lung Carcinoma (LLC) from National Cancer Institute were grown in DMEM supplemented with 10% FBS, 1% L-Glutamine, and 1% Penicillin/Streptomycin. THP-1 from ATCC, MOLM13 from Dr. Lee Grimes (Cincinnati Children’s Hospital), and OCI-AML2 from Dr. Suming Huang (University of Florida) were grown in RPMI 1640 supplemented with 10% FBS, 1% L-Glutamine, and 1% Penicillin/Streptomycin. HEK293T from ATCC was grown in DMEM supplemented with 10% BCS, 1% L-Glutamine, and 1% Penicillin/Streptomycin. All cell lines were cultured in an incubator with 5% CO_2_ at 37°C and tested negative for *Mycoplasma* contamination.

CD11b^+^ myeloid cells were isolated from bone marrow by using CD11b microbeads (Miltenyi Biotec). T cells were isolated from spleen by using Dynabeads^™^ Untouched^™^ Mouse T Cells Kit (Invitrogen). Murine peritoneal macrophages were harvested 4 days after peritoneal injection of 4% thioglycollate (BD). Bone marrow-derived macrophages (BMDMs) were generated by culturing total bone marrow cells from femurs and tibiae in RPMI 1640 and 10% FBS supplemented with 30 ng/ml recombinant murine CSF1 (Peprotech) for 7 days. 40 ng/ml IL-4 (Peprotech), 20 ng/ml IL-13 (Peprotech), and 20 ng/ml CSF1 were added for 24 hours for polarization towards immunosuppressive macrophages. PLX3397 (Selleckchem) and GYY4137 (abcam) were used for inhibition of CSF1R and NF-κB, respectively. For *in vitro* deletion of Usp18, total bone marrow cells from *Usp18*^*f/f*^ *UBC-Cre-ERT2* mice were treated with β-estradiol (2 μM) for 48 hours. THP-1-derived macrophages were generated by culturing in the media containing PMA (50 ng/ml) for 48 hours. hUSP18 knockout THP-1 line was generated with CRISPR genome editing technology as previously described.^19^ sgRNA-resistant wild-type or C64S mutant hUSP18 was expressed in the hUSP18 knockout THP-1 cells.

For interferon treatment, mouse IFN-β (PBL Assay Science) and Universal Type I IFN (R&D Systems) were used for murine and human cell lines, respectively. For the analysis of downstream signaling of CSF1R, cells were treated with IFN-β (100 U/ml) for 24 hours followed by CSF1 (50 ng/ml) for 5 or 10 minutes after 4-hour starvation. For a protein stability assay, cells were treated with cycloheximide (CHX) at 50 μg/ml. Lactacystin (10 μM) was used as a proteasome inhibitor.

### METHOD DETAILS

#### Genomic DNA extraction and PCR

Genomic DNA was extracted from tail with Allele-In-One Mouse Tail Direct Lysis Buffer (Allele Biotechnology) or from macrophage with TRIzol (Invitrogen). PCR was performed with Taq or Pfu enzymes generated in our lab.

#### Reverse transcription and quantitative PCR

Total RNA was extracted using TRIzol (Invitrogen) and reverse transcribed with First Strand cDNA Synthesis Kit (MCLAB) according to the manufacturer’s instructions. Quantitative PCR analysis was performed by using KAPA SYBR FAST (KAPA Biosystems) on the CFX96 thermal cycler (BIO-RAD). Primer sequences are listed in the key resources table.

#### Plasmid construction

m*Usp18*, h*USP18* and its mutants were cloned into pCX4-bsr retroviral vector. m*Csf1r* and h*CSF1R* cDNA were cloned into pCAG vector. Ubiquitin, m*Nedd4* and its mutants, and h*NEDD4* cDNAs were cloned into pcDNA3 vector. h*CSF1R* cytoplasmic domain (543 – 972) and h*NEDD4* were cloned into pGEX-6P-1 (cytiva). shRNA for h*USP18* was cloned into pSUPER.retro.puro vector (OligoEngine). GFP was cloned into pLKO.1 vector as a negative control for shRNA knockdown experiment. All the constructs were confirmed by DNA sequencing. shRNA pLKO.1 vectors for m*Nedd4* (TRCN0000092436), h*NEDD4* (TRCN0000007550, TRCN0000007551), and h*UBCH5C* (TRCN0000038791, TRCN0000038793) were purchased from La Jolla Institute for Immunology.

#### Transfection and infection

Transfection was performed by using polyethylenimine (PEI). For retrovirus and lentivirus infection, HEK293T cells were co-transfected with plasmids encoding target genes and packaging vectors; pCL-10A1 or pCL-Eco for retrovirus, psPAX2 and pMD2.G for lentivirus. Viral particles from culture medium were collected and infected to target cells by spin infection (2,000 × g, 3 h, 30 °C) with hexadimethrine bromide (8 μg/ml). Infected cells were selected with appropriate selection drugs.

#### Immunoprecipitation and western blotting

Cells were lysed in lysis buffer composed of 25 mmol/L Tris-HCl, pH 8.0, 150 mmol/L NaCl, 1 mmol/L EDTA, 0.5% IgepalCA-630, and protease/phosphatase inhibitors (Roche). The cell lysates were centrifuged (20,000 × g) at 4 °C for 5 minutes. For co-immunoprecipitation assay, cell lysates were immunoprecipitated for 1 to 2 hours with FLAG M2 Affinity Gel for FLAG-tagged proteins or with primary antibodies as indicated followed by protein G/A-Agarose Suspension (EMD Millipore) for other proteins. Immunocomplexes were then adsorbed to the protein G/A-Agarose Suspension and washed three times. All samples were denatured in 1x sample buffer (50 mmol/L Tris-HCl, pH 6.8, 2% SDS, 5% 2-mercaptoethanol, 10% glycerol, and 0.01% bromophenol blue) for 5 minutes at 100 °C.

Proteins were electroblotted onto nitrocellulose membranes (cytiva) and incubated with primary antibodies. Li-Cor Fluorophoreconjugated secondary antibodies (Li-Cor) were used for detection by Odyssey system (Li-Cor). The following primary antibodies were used; anti-CSF1R, anti-NEDD4, anti-UBCH5C, anti-Akt, anti-Phospho-Akt, anti-p38, anti-Phospho-p38, anti-SAPK/JNK, and anti-Phospho-SAPK/JNK from Cell Signaling Technology, anti-β-actin, anti-α-tubulin, and anti-FLAG M2 from Sigma-Aldrich, anti-Myc and anti-Ubiquitin from Santa Cruz Biotechnology, anti-HA from Roche, anti-GFP from Invitrogen. Anti-ISG15 and anti-USP18 antibodies were previously described.^14,15^ Quantification was performed with LI-COR Image Studio software.

#### In vitro ubiquitination assay

GST-CSF1R cytoplasmic domain (543 – 972) substrate protein and GST-NEDD4 E3 ligase were purified from *E. coli* BL21 by Glutathione Sepharose 4B (cytiva). GST was cleaved from NEDD4 by Prescission protease (cytiva). *In vitro* ubiquitination reactions were performed using Ubiquitinylation kit (Enzo Life Sciences) according to the manufacturer’s instructions. Briefly, Glutathione Sepharose 4B only, retained GST, or GST-CSF1R was incubated with UBE1, E2, and E3 enzymes in a buffer containing ATP and ubiquitin at 30°C for 1 hour. The GST-CSF1R were eluted from the resins and were subjected to western blot analysis.

#### Flow cytometry analysis

Total bone marrow cells from *Usp18*^*f/f*^ *UBC-Cre-ERT2* mice were harvested and cultured with or without β-estradiol (2 μM) for 48 hours. Cells were then stained with anti-CD11b antibody and propidium iodide for cell viability assay.

For immunophenotyping of tumor-bearing mice, single-cell suspension was prepared from tumor tissue and tumor-draining lymph nodes by passing through a 40 μm cell strainer. Single-cell suspension was then treated with ACK buffer for RBC lysis, and density gradient centrifugation on 40%/80% Percoll (cytiva) gradient was performed. After Fc blocking with anti-CD16/CD32 (eBioscience), cells were stained and analyzed on a BD FACS Canto II, BD LSR II, or Agilent NovoCyte Advanteon with standard lasers and optical filters. For single-cell RNA sequencing analysis, cells were sorted by BD FACS Aria II with standard lasers and optical filters. Fluorochrome-conjugated primary antibodies used in the study are listed in the key resources table. Propidium Iodide or Fixable Zombie (BioLegend) was used for viability staining. Foxp3/Transcription Factor Staining Buffer set (eBioscience) was used for fixation and permeabilization. Data were analyzed on FlowJo (FlowJo, LLC).

#### Single-cell RNA sequencing and analysis

Single-cell suspension of B16F10 melanoma from *Usp18*^*f/f*^ *or Usp18*^*f/f*^ *LysM-Cre* was prepared as described above. Cells were pooled from 3 mice (*Usp18*^*f/f*^) or 4 mice (*Usp18*^*f/f*^ *LysM-Cre*). CD45^+^ cells were then sorted on BD FACS Aria. Cells were counted with Countess II FL (Invitrogen) and loaded onto the 10x Genomics Chromium controller. Libraries were prepared using Chromium Single Cell 3’Reagent Kit v3 (10x Genomics) as per the manufacturer’s protocol. The generated libraries were sequenced using an Illumina HiSeq 4000 at the Institute for Genomic Medicine at University of California, San Diego. Sequencing data were aligned using the 10x Genomics Cell Ranger pipeline^83^ (v3.0.1, mm10 reference genome) and subsequently analyzed using Seurat v4.3.0.^84^

For cell type annotation, an automated method with manual modification was employed. The clusters were first annotated with SingleR^28^ using prebuilt ImmGen database^29^ reference and then modified based on the gene expression and TAM classification from a published article^30^ as needed.

Pathway enrichment analysis was performed with Gene Set Enrichment Analysis (GSEA).^31,32^ First, differentially expressed genes (DEGs) were obtained by pesudobulk gene expression analysis^87^ using the DEseq2^86^ model. Differentially expressed genes of each cluster were pre-ranked by differential test-statistic and analyzed by GSEA using the H: Hallmarks and C2: canonical pathways geneset collections. Metascape^33^ was also used for pathway enrichment analysis. For trajectory analysis, differentially expressed genes of TAM clusters (Cluster 1, 2, 6, 7, and 12) were analyzed by Monocle 3 using default and developer-recommended settings.^41–43^

#### Bulk RNA sequencing and analysis

RNA was extracted from untreated or IFN-I-treated (1000 U/ml for 6 hours) THP-1 WT and USP18^−/−^ THP-1 cells by using TRIzol re-agent. All RNA-seq libraries were prepared by Novogene and sequenced using an Illumina Novaseq 6000 (PE150). STAR^85^ was used for alignment. Differential gene expression analysis was performed using the DESeq2.^86^ Genes with an adjusted *P*-value <0.05 were considered as differentially expressed.

### QUANTIFICATION AND STATISTICAL ANALYSIS

#### Statistical analysis

Experiments were replicated two to four times. Data are presented as the means with S.D. or S.E.M. as indicated. Statistical significance was determined by Prism 8 (GraphPad). Two-tailed unpaired t-test and ordinary one-way ANOVA multiple comparison test with post-hoc Tukey test were conducted for comparisons of two groups and more than two groups, respectively. *P value p* <0.05 was considered to be statistically significant. *; *p* < 0.05, **; *p* < 0.01, ***; *p* < 0.001, ****; *p* < 0.0001.

## Supplementary Material

1

## Figures and Tables

**Table:** KEY RESOURCES TABLE

REAGENT or RESOURCE	SOURCE	IDENTIFIER
Antibodies		
InVivoMAb anti-mouse IFNAR-1 (Clone MAR1-5A3)	Bio X Cell	Cat #: BE0241; RRID: AB_2687723
InVivoMAb mouse IgG1 isotype control (Clone MOPC-21)	Bio X Cell	Cat #: BE0083; RRID: AB_1107784
Rat monoclonal anti-mouse CD16/CD32 (Clone: 93)	Thermo Fisher Scientific (eBioscience)	Cat #: 14-0161-82; RRID: AB_467133
Mouse monoclonal anti-mouse CD45.2 (clone 104) Brilliant Violet 605	BioLegend	Cat #: 109841; RRID: AB_2563485
Mouse monoclonal anti-mouse CD45.2 (clone 104) PerCP-Cyanine5.5	BioLegend	Cat #: 109828; RRID: AB_893350
Mouse monoclonal anti-mouse CD45.2 (clone 104) Alexa Fluor 700	BioLegend	Cat #: 109822; RRID: AB_493731
Rat monoclonal anti-mouse/human CD11b (clone M1/70) Alexa Fluor 700	BD Biosciences	Cat #: 557960; RRID: AB_396960
Rat monoclonal anti-mouse/human CD11b (clone M1/70) FITC	BD Biosciences	Cat #: 553310; RRID: AB_394774
Rat monoclonal anti-mouse/human CD11b (clone M1/70) PE-Cyanine7	BioLegend	Cat #: 101216; RRID: AB_312799
Armenian Hamster monoclonal anti-mouse CD11c (clone N418) Pacific Blue	BioLegend	Cat #: 117322; RRID: AB_755988
Rat monoclonal anti-mouse F4/80 (clone BM8) Brilliant Violet 605	BioLegend	Cat #: 123133; RRID: AB_2562305
Rat monoclonal anti-mouse F4/80 (clone BM8) APC	BioLegend	Cat #: 123116; RRID: AB_893481
Rat monoclonal anti-mouse Gr-1 (Ly6G/Ly6C) (clone RB6-8C5) PE-Cyanine7	BD Biosciences	Cat #: 565033; RRID: AB_2739049
Rat monoclonal anti-mouse CD115 (CSF-1R) (clone AFS98) APC	BioLegend	Cat #: 135510; RRID: AB_2085221
Rat monoclonal anti-mouse CD115 (CSF-1R) (clone AFS98) PE	BioLegend	Cat #: 135506; RRID: AB_1937253
Rat monoclonal anti-mouse CD3 (clone 17A2) Brilliant Violet 510	BioLegend	Cat #: 100234; RRID: AB_2562555
Armenian Hamster monoclonal anti-mouse CD3e (clone 145-2C11) Brilliant Violet 650	BD Biosciences	Cat #: 564378; RRID: AB_2738779
Rat monoclonal anti-mouse CD4 (clone RM4-5) Pacific Blue	BD Biosciences	Cat #: 558107; RRID: AB_397030
Rat monoclonal anti-mouse CD4 (clone RM4-5) Brilliant Violet 570	BioLegend	Cat #: 100542; RRID: AB_2563051
Rat monoclonal anti-mouse CD8a (clone 5H10) Pacific Orange	Thermo Fisher Scientific (Invitrogen)	Cat #: MCD0830; RRID: AB_10376311
Rat monoclonal anti-mouse CD8a (clone 53-6.7) PE-Cyanine5	BioLegend	Cat #: 100710; RRID: AB_312749
Rat monoclonal anti-mouse Ly6G (clone 1A8) APC-Cyanine7	BioLegend	Cat #: 127623; RRID: AB_10645331
Rat monoclonal anti-mouse Ly6C (clone HK1.4) PE-Dazzle594	BioLegend	Cat #: 128043; RRID: AB_2566576
Mouse monoclonal anti-mouse NK-1.1 (clone PK136) PE	BioLegend	Cat #: 108708; RRID: AB_313395
Rat monoclonal anti-mouse/human CD45R/B220 (clone RA3-6B2) Alexa Fluor 700	BioLegend	Cat #: 103232; RRID: AB_493717
Rat monoclonal anti-mouse CD25 (clone PC61) PE-Cyanine7	BioLegend	Cat #: 102016; RRID: AB_312865
Rat monoclonal anti-mouse Foxp3 (clone MF23) Alexa Fluor 647	BD Biosciences	Cat #: 560401; RRID: AB_1645201
Rat monoclonal anti-mouse Foxp3 (clone FJK-16s) APC	Thermo Fisher Scientific (eBioscience)	Cat #: 17-5773-82; RRID: AB_469457
Rat monoclonal anti-mouse/human CD44 (clone IM7) FITC	BioLegend	Cat #: 103006; RRID: AB_312957
Rat monoclonal anti-mouse CD62L (clone MEL-14) PerCP-Cyanine5.5	BioLegend	Cat #: 104432; RRID: AB_2285839
Armenian Hamster monoclonal anti-mouse CD69 (clone H1.2F3) PE-Dazzle594	BioLegend	Cat #: 104536; RRID: AB_2565583
Rat monoclonal anti-mouse IFN-γ (clone XMG1.2) PE	BioLegend	Cat #: 505807; RRID: AB_315401
Rat monoclonal anti-mouse Perforin (clone S16009A) PE-Dazzle594	BioLegend	Cat #: 154315; RRID: AB_2922482
Rat monoclonal anti-mouse CD206 (MMR) (clone C068C2) PE	BioLegend	Cat #: 141705; RRID: AB_10896421
Rabbit polyclonal anti-CSF-1R/M-CSF-R	Cell Signaling Technology	Cat #: 3152; RRID: AB_2085233
Rabbit monoclonal anti-CSF-1R/M-CSF-R (clone D3O9X)	Cell Signaling Technology	Cat #: 67455; RRID: AB_2799725
Rabbit polyclonal anti-NEDD4	Cell Signaling Technology	Cat #: 2740; RRID: AB_2149312
Rabbit monoclonal anti-UbcH5C (clone D60E2)	Cell Signaling Technology	Cat #: 4330; RRID: AB_10544697
Rabbit polyclonal anti-Akt	Cell Signaling Technology	Cat #: 9272; RRID: AB_329827
Rabbit monoclonal anti-Phospho-Akt (Ser473) (clone D9E)	Cell Signaling Technology	Cat #: 4060; RRID: AB_2315049
Rabbit polyclonal anti-p38 MAPK	Cell Signaling Technology	Cat #: 9212; RRID: AB_330713
Rabbit monoclonal anti-Phospho-p38 (Thr180/Tyr182) (clone 12F8)	Cell Signaling Technology	Cat #: 4631; RRID: AB_331765
Rabbit monoclonal anti-SAPK/JNK (clone 56G8)	Cell Signaling Technology	Cat #: 9258; RRID: AB_2141027
Rabbit polyclonal anti-Phospho-SAPK/JNK (Thr183/Tyr185)	Cell Signaling Technology	Cat #: 9251; RRID: AB_331659
Mouse monoclonal anti-β-actin (clone AC-15)	Sigma-Aldrich	Cat #: A1978; RRID: AB_476692
Mouse monoclonal anti-α-tubulin (clone DM1A)	Sigma-Aldrich	Cat #: T6199; RRID: AB_477583
Mouse monoclonal anti-FLAG M2	Sigma-Aldrich	Cat #: F3165; RRID: AB_259529
Mouse monoclonal anti-Myc (clone 9E10)	Santa Cruz Biotechnology	Cat #: sc-40; RRID: AB_627268
Rat monoclonal anti-HA (clone 3F10)	Roche	Cat #: 11867423001; RRID: AB_390918
Rabbit polyclonal anti-GFP	Thermo Fisher Scientific (Invitrogen)	Cat#: A-11122; RRID: AB_221569
Anti-ISG15	Malakhov et al.^14^	N/A
Anti-USP18	Malkahova et al.^15^	N/A
Chemicals, peptides, and recombinant proteins		
BD DIFCO^™^ Thioglycollate Medium, Brewer Modified	Becton Dickinson	Cat#: 211716
Recombinant Murine M-CSF	Peprotech	Cat#: 315-02
Recombinant Murine IL-4	Peprotech	Cat#: 214-14
Recombinant Murine IL-13	Peprotech	Cat#: 210-13
PMA (Phorbol 12-Myristate 13-Acetate)	Sigma-Aldrich	Cat#: P1585
Mouse IFN-Beta, Mammalian	PBL Assay Science	Cat#: 12405
Universal Type I IFN Protein	R&D Systems	Cat#: 11200-2
Cycloheximide	Sigma-Aldrich	Cat#: 01810
Lactacystin	Sigma-Aldrich	Cat #: L6785
β-Estradiol	Sigma-Aldrich	Cat #: E2758
Polyinosinic-polycytidylic acid sodium salt	Sigma-Aldrich	Cat#: P0913
Pexidartinib (PLX3397)	Selleckchem	Cat#: S7818
GYY4137, H2S donor	abcam	Cat#: ab142145
Percoll^™^	cytiva	Cat #: 17089101
Propidium Iodide	Roche	Cat#: 11348639001
eBioscience^™^ Foxp3 / Transcription Factor Staining Buffer Set	Thermo Fisher Scientific (Invitrogen)	Cat #: 00–5523-00
Allele-In-One Mouse Tail Direct Lysis Buffer	Allele Biotechnology	Cat #: ABP-PP-MT01500
TRIzol^™^ Reagent	Thermo Fisher Scientific (Invitrogen)	Cat#: 15596018
First Strand cDNA Synthesis Kit	MCLAB	Cat #: FSCS-200
KAPA SYBR FAST qPCR Kit Master Mix (2X) Universal	KAPA Biosystems	Cat #: KK4602
cOmplete^™^, EDTA-free Protease Inhibitor Cocktail	Sigma-Aldrich (Roche)	Cat#: 11873580001
PhosSTOP^™^	Sigma-Aldrich (Roche)	Cat #: 4906837001
Protein G Plus/Protein A Agarose Suspension	EMD Millipore	Cat #: IP05
Anti-FLAG M2 Affinity Gel	Sigma-Aldrich	Cat #: A2220
PEI (Polyethylenimine, branched)	Sigma-Aldrich	Cat #: 408727
Hexadimethrine bromide	Sigma-Aldrich	Cat #: H9268
Glutathione Sepharose 4B	cytiva	Cat #: 17075601
PreScission Protease	cytiva	Cat #: 27084301
Critical commercial assays		
CD11b MicroBeads, human and mouse	Miltenyi Biotec	Cat #: 130-049-601
Dynabeads^™^ Untouched^™^ Mouse T Cells Kit	Invitrogen	Cat#: 11413D
Chromium Single Cell 3’Reagent Kit v3	10x Genomics	Cat#: PN-1000075
Zombie Aqua^™^ Fixable Viability Kit	BioLegend	Cat#: 423101
Zombie NIR^™^ Fixable Viability Kit	BioLegend	Cat#: 423106
Ubiquitinylation kit	Enzo Life Sciences	Cat #: BML-UW9920-0001
Deposited data		
Single-cell RNAseq data	This paper	GEO: GSE173705
Bulk RNAseq data	Arimoto et al.^19^	GEO: GSE165428
Experimental models: Cell lines		
Mouse: B16F10	Dr. Michiko Fukuda (Sanford Burnham Prebys Institute)	RRID: CVCL_0159
Mouse: EL4	American Type Culture Collection (ATCC)	Cat #: TIB-39; RRID: CVCL_0255
Mouse: Lewis Lung Carcinoma (LLC)	National Cancer Institute	RRID: CVCL_4358
Human: THP-1	ATCC	Cat #: TIB-202; RRID: CVCL_0006
Human: THP-1 USP18 knockout	Arimoto et al.^19^	N/A
Human: MOLM13	Dr. Lee Grimes (Cincinnati Children’s Hospital)	RRID:CVCL_2119
Human: OCI-AML2	Dr. Suming Huang (University of Florida)	RRID:CVCL_1619
Human: HEK293T	ATCC	Cat #: CRL-3216; RRID: CVCL_0063
Experimental models: Organisms/strains		
Mouse: B6.129P2-*Lyz2*^*tm1(cre)Ifo*^/J	The Jackson Laboratory	Strain #: 004781; RRID: IMSR_JAX:004781
Mouse: B6.Cg-^*Ndor1Tg(UBC-cre/ERT2)1Ejb*^/1J	The Jackson Laboratory	Strain #: 008085; RRID: IMSR_JAX:008085
Mouse: *Usp18*^*f/f*^	Arimoto et al.^19^	N/A
Mouse: *Isg15* knockout	Osiak et al.^82^	N/A
Oligonucleotides		
Primer: mouse *Gapdh* Forward: TATGTCGTGGAGTCTACTGG	This paper	N/A
Primer: mouse *Gapdh* Reverse: GAGTTGTCATATTTCTCGTG	This paper	N/A
Primer: mouse *Usp18* Forward: CAGCCCTCATGGTCTGGTTG	This paper	N/A
Primer: mouse *Usp18* Reverse: GCACTCCGAGGCACTGTTAT	This paper	N/A
Primer: mouse *Csf1r* Forward: AATGCTAACGCCACCGAGA	This paper	N/A
Primer: mouse *Csf1r* Reverse: CATGGAAAGTTCGGACACAGG	This paper	N/A
Primer: mouse *Nedd4* Forward: ACCAGCGTGCAGACAAAAAC	This paper	N/A
Primer: mouse *Nedd4* Reverse: AAAAGAATGCGGTGTCGCTG	This paper	N/A
Recombinant DNA		
Plasmid: pCL-10A1	Novus	Cat #: NBP2-29542
Plasmid: pCL-Eco	Novus	Cat #: NBP2-29540
Plasmid: psPAX2	Dr. Didier Trono (Addgene)	RRID: Addgene_12260
Plasmid: pMD2.G	Dr. Didier Trono (Addgene)	RRID: Addgene_12259
Plasmid: pCX4-bsr	Dr. Tsuyoshi Akagi (KAN Research Institute)	N/A
Plasmid: pcDNA^™^3.1 (+) Mammalian Expression Vector	Thermo Fisher Scientific (Invitrogen)	Cat #: V79020
Plasmid: pCX4-bsr-mouse Usp18	This paper	N/A
Plasmid: pCX4-bsr -FLAG-mouse Usp18	This paper	N/A
Plasmid: pCX4-bsr-human USP18 (sgRNA resistant)	This paper	N/A
Plasmid: pCX4-bsr-human USP18 C64S (sgRNA resistant)	This paper	N/A
Plasmid: pCAG-mouse Csf1r-FLAG	This paper	N/A
Plasmid: pCAG-human CSF1R-FLAG	This paper	N/A
Plasmid: pcDNA-Myc-human NEDD4	This paper	N/A
Plasmid: pcDNA -HA-mouse Nedd4	This paper	N/A
Plasmid: pcDNA -HA-mouse Nedd4 (C744A mutant)	This paper	N/A
Plasmid: pcDNA -HA-mouse Nedd4 (HECT domain deletion mutant)	This paper	N/A
Plasmid: pcDNA-FLAG-mouse Nedd4	This paper	N/A
Plasmid: pcDNA-Myc-Ubiquitin	This paper	N/A
Plasmid: pEGFP-N1	Clontech	Cat #: 6085-1
Plasmid: pSUPER.retro.puro	OligoEngine	Cat #: VEC-PRT-0002
Plasmid: pSUPER.retro.puro-human USP18 shRNA	This paper	N/A
Plasmid: pLKO.1 Negative control (GFP)	This paper	N/A
Plasmid: pLKO.1-human UBCH5C shRNA	La Jolla Institute for Immunology	Cat #: TRCN0000038791
Plasmid: pLKO.1-human UBCH5C shRNA	La Jolla Institute for Immunology	Cat #: TRCN0000038793
Plasmid: pLKO.1-mouse Nedd4 shRNA	La Jolla Institute for Immunology	Cat #: TRCN0000092436
Plasmid: pLKO.1-human NEDD4 shRNA	La Jolla Institute for Immunology	Cat #: TRCN0000007550
Plasmid: pLKO.1-human NEDD4 shRNA	La Jolla Institute for Immunology	Cat #: TRCN0000007551
Plasmid: pGEX-6P-1	cytiva	Cat #: 28954648
Plasmid: pGEX-6P-1-human CSF1R (543 – 972)	This paper	N/A
Plasmid: pGEX-6P-1-human NEDD4	This paper	N/A
Software and algorithms		
GraphPad Prism 8	GraphPad	N/A
FlowJo 10	FlowJo, LLC	N/A
LI-COR Image Studio^™^ Software	LI-COR	N/A
10x Genomics Cell Ranger (v3.0.1)	Zheng et al.^83^	N/A
Seurat (v4.3.0)	Hao et al.^84^	https://satijalab.org/seurat/
STAR	Dobin et al.^85^	N/A
DESeq2	Love et al.^86^	N/A
SingleR	Aran et al.^28^	N/A
ImmGen (Immunological Genome Project)	Heng et al.^29^	N/A
Gene Set Enrichment Analysis (GSEA)	Mootha et al.^31^ Subramanian et al.^32^	https://www.gsea-msigdb.org
Metascape	Zhou et al.^33^	https://metascape.org
Monocle 3	Cao et al.^41^ Qiu et al.^42^ Trapnell et al.^43^	N/A
Other		
scil Vet abc Plus hematology analyzer	scil animal care company	N/A
MycoAlert PLUS Mycoplasma Detection Kit	Lonza	Cat#: LT07-710
BD FACS Canto II Flow Cytometer	BD Biosciences	N/A
BD LSR II Flow Cytometer	BD Biosciences	N/A
BD FACS Aria II Cell Sorter	BD Biosciences	N/A
NovoCyte Advanteon Flow Cytometer	Agilent Technologies	N/A
CFX96 Thermal cycler	Bio-Rad Laboratories	N/A
Odyssey Imaging System	LI-COR Biotechnology	N/A
Countess II FL Automated Cell Counter	Thermo Fisher Scientific (Invitrogen)	N/A
Chromium controller	10x Genomics	N/A

## Highlights

- Deletion of USP18 in myeloid cells suppresses tumor progression
- Deletion of USP18 increases anti-tumor macrophages in the tumor microenvironment
- Deletion of USP18 in myeloid cells induces downregulation of CSF1R in macrophages
- USP18 impairs degradation of CSF1R by interrupting the binding of E3 ligase NEDD4
