# Supplementary material for: Reprogramming of tumor-associated macrophages via NEDD4-mediated CSF1R degradation by targeting USP18
Source: Cell Rep. Author manuscript; Available in PMC 2024 Jan 28. (PMC10822669; doi:10.1016/j.celrep.2023.113560)
Supplement: 1 [file NIHMS1954800-supplement-1.pdf]

Cell Reports, Volume *42*

Supplemental information

Reprogramming of tumor-associated macrophages via NEDD4-mediated CSF1R degradation by targeting USP18

Sayuri Miyauchi, Kei-ichiro Arimoto, Mengdan Liu, Yue Zhang, and Dong-Er Zhang

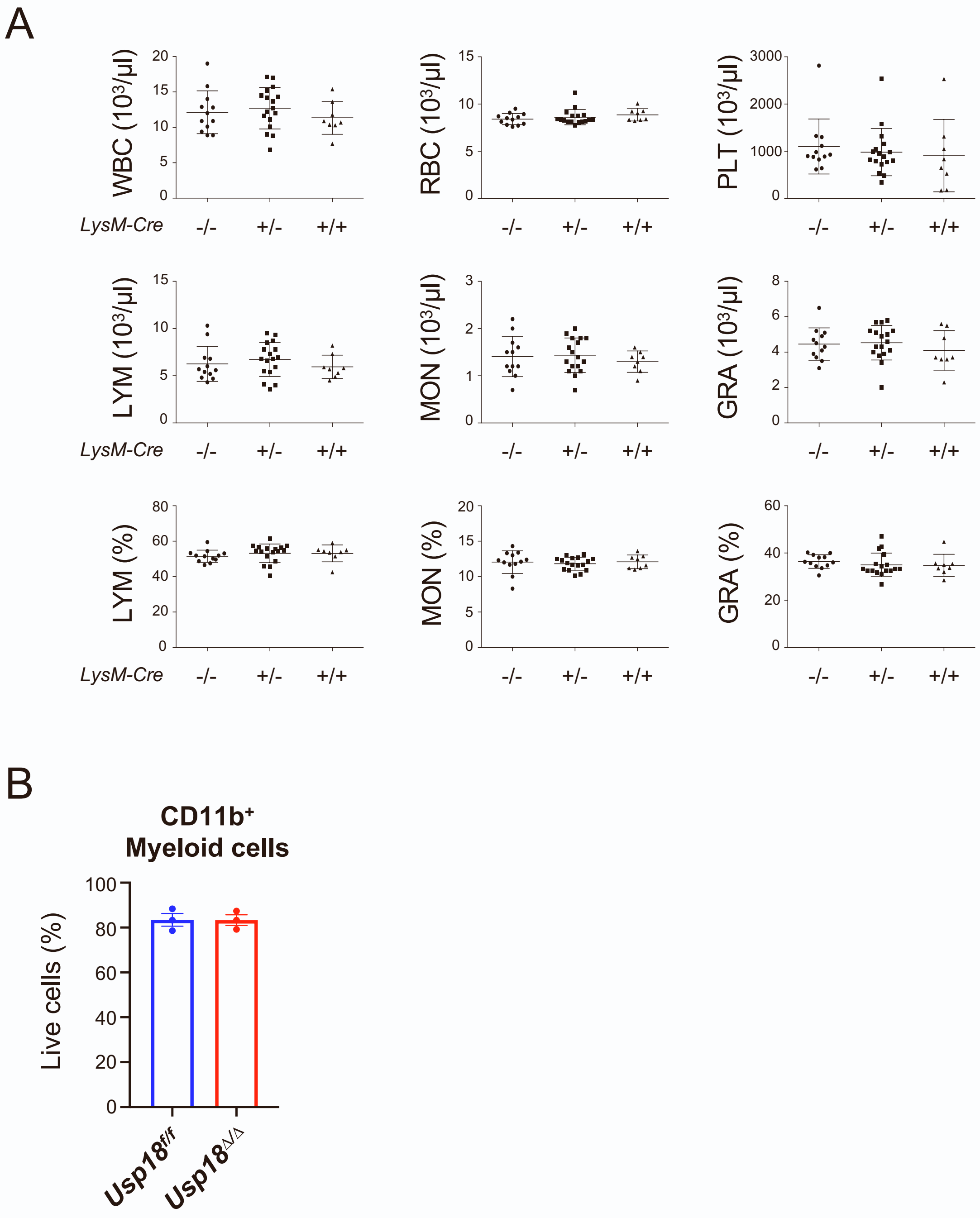

Figure S1, Related to Figure 1. Normal hematopoiesis in *Usp18*$^{\Delta/\Delta}$ mice

(A) Hematological analysis of peripheral blood from *Usp18*$^{f/f}$ *LysM-Cre*$^{-/-}$ (n = 12), *Usp18*$^{f/f}$ *LysM-Cre*$^{+/-}$ (n = 17) or *Usp18*$^{f/f}$ *LysM-Cre*$^{+/+}$ mice (n = 8). Mean ± S.D. WBC; white blood cells, RBC; red blood cells, PLT; platelets, LYM; lymphocytes, MON; monocytes, GRA; granulocytes.

(B) The total bone marrow cells were harvested from *Usp18*$^{f/f}$ *UBC-Cre-ERT2* mice and the deletion of *Usp18* was induced by β-estradiol treatment *in vitro*. After 48 hours, the cell viability of CD11b^{+} myeloid cells was analyzed by flow cytometry (n = 3). Mean ± S.E.M.

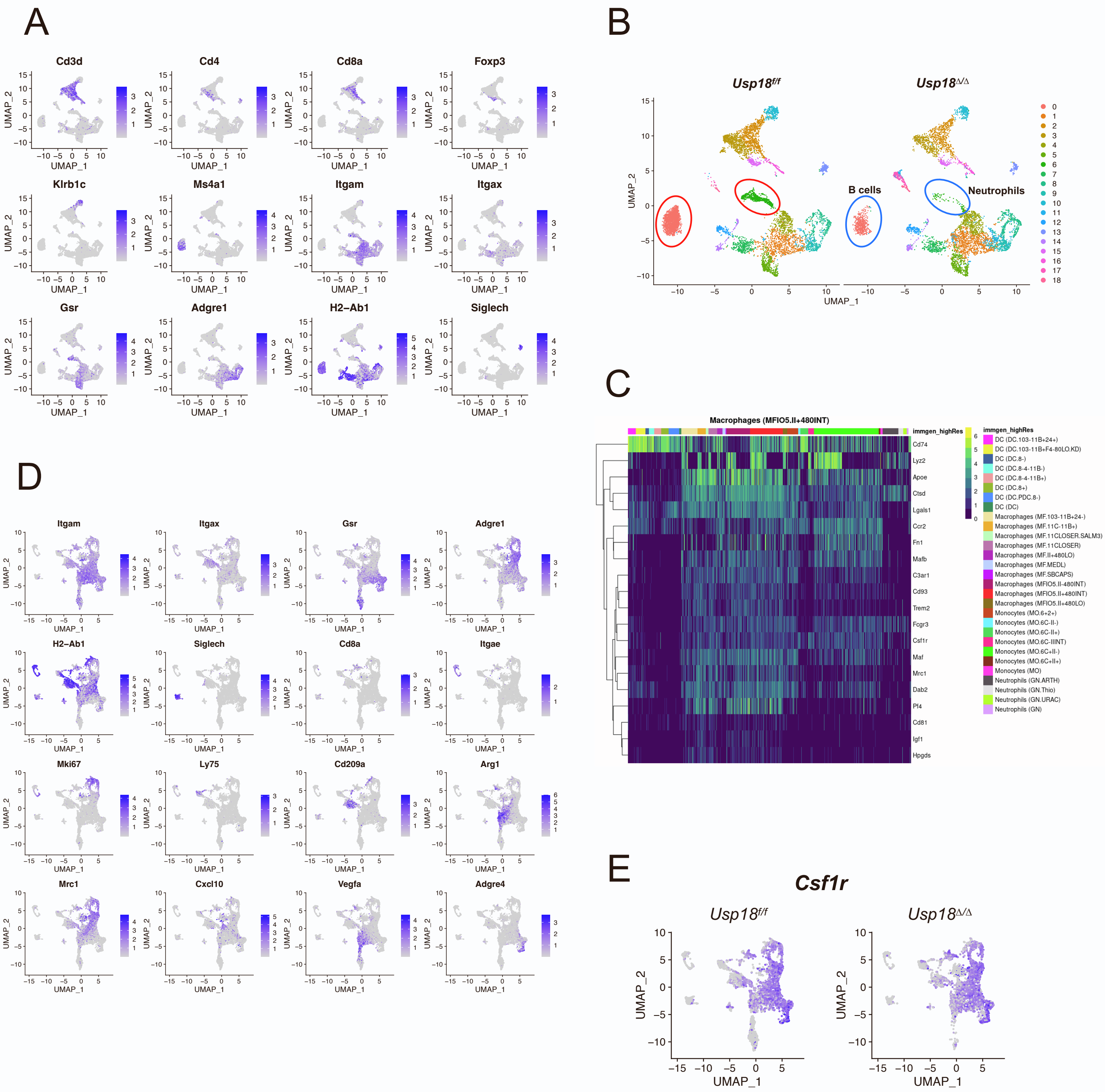

Figure S2, Related to Figure 3. Single-cell analysis of tumor-infiltrating immune cells

(A) Feature plots of intratumoral CD45^{+} cells from merged sample.

(B) UMAP plot of intratumoral CD45^{+} cells split by *Usp18$^{f/f}$* and *Usp18$^{\Delta/\Delta}$* mice.

(C) A representative cell type and supporting marker gene heatmap generated by the automated annotation program SingleR.

(D) Feature plots of monocytes/macrophages/dendritic cells/neutrophils subpopulation (Mono/Mac/DC/Neu).

(E) Expression of *Csf1r* in *Usp18$^{f/f}$* and *Usp18$^{\Delta/\Delta}$* mice.

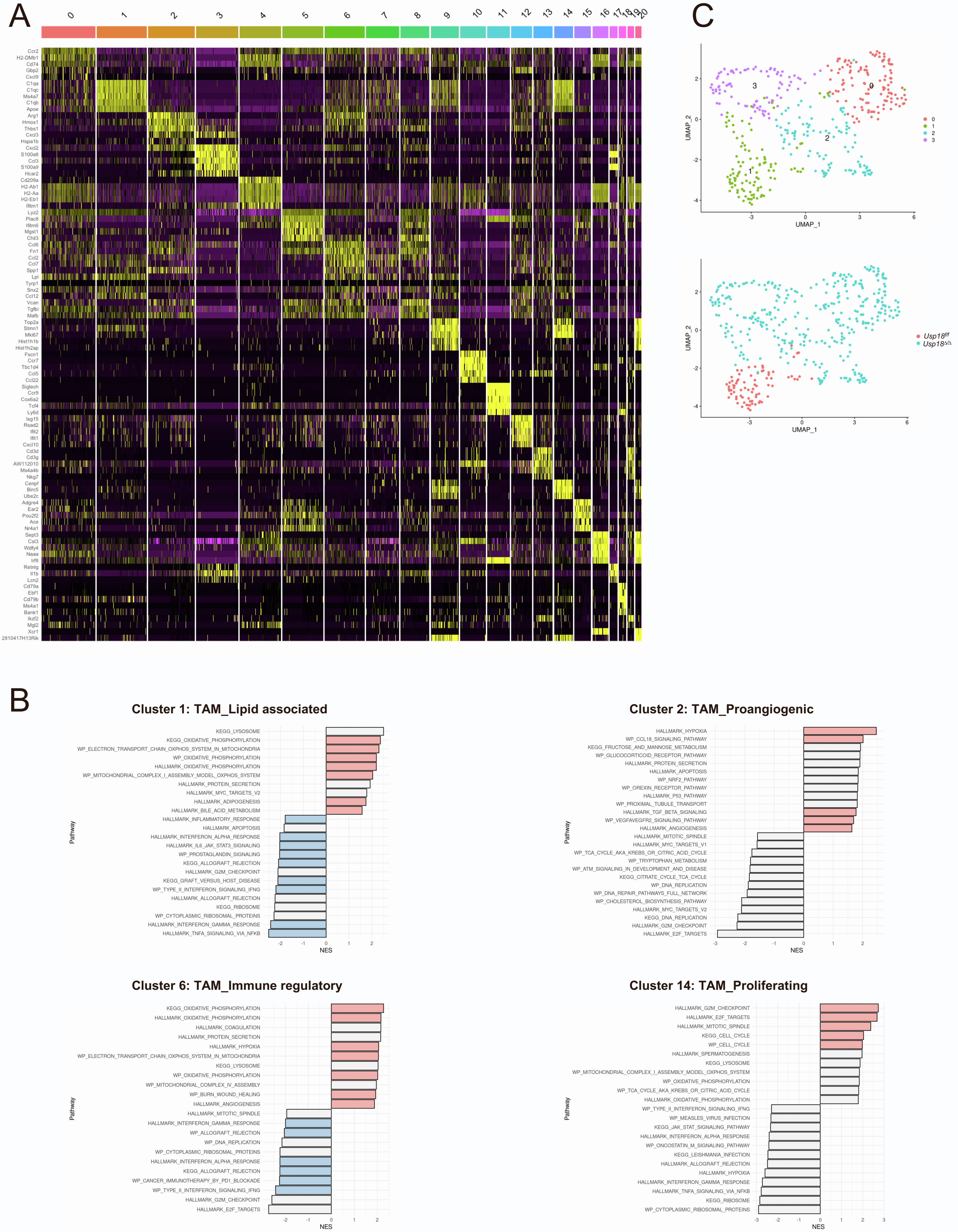

A
0
1
2
3
4
5
6
7
8
9
10
11
12
13
14
15
16
17
18
19
20
Ccr2
H2-DMb1
Cd74
Gbp2
Cxcl9
C1qa
C1qc
Ms4a7
C1qb
Apoe
Arg1
Hmox1
Thbs1
Cxcl3
Hspa1b
Cxcl2
S100a8
Ccl3
S100a9
Hcar2
Cd209a
H2-Ab1
H2-Aa
H2-Eb1
Ifitm1
Lyz2
Plac8
Ifitm6
Mgst1
Chil3
Ccl6
Fn1
Ccl2
Ccl7
Spp1
Lpl
Tyrp1
Snx2
Ccl12
Vcan
Tgfbi
Mafb
Top2a
Stmn1
Mki67
Hist1h1b
Hist1h2ap
Fscn1
Ccr7
Tbc1d4
Ccl5
Ccl22
Siglech
Ccr9
Cox6a2
Tcf4
Ly6d
Isg15
Rsad2
Ifit2
Ifit1
Cxcl10
Cd3d
Cd3g
AW112010
Ms4a4b
Nkg7
Cenpf
Birc5
Ube2c
Adgre4
Ear2
Pou2f2
Ace
Nr4a1
Sept3
Cst3
Wdfy4
Naaa
Irf8
Retnlg
Il1b
Lcn2
Cd79a
Ebf1
Cd79b
Ms4a1
Bank1
Ikzf2
Mgl2
Xcr1
2810417H13Rik
C
UMAP_2
UMAP_1
0
1
2
3
Usp18f/f
Usp18ΔΔ
B
Cluster 1: TAM_Lipid associated
KEGG_LYSOSOME
KEGG_OXIDATIVE_PHOSPHORYLATION
WP_ELECTRON_TRANSPORT_CHAIN_OXPHOS_SYSTEM_IN_MITOCHONDRIA
WP_OXIDATIVE_PHOSPHORYLATION
HALLMARK_OXIDATIVE_PHOSPHORYLATION
WP_MITOCHONDRIAL_COMPLEX_I_ASSEMBLY_MODEL_OXPHOS_SYSTEM
HALLMARK_PROTEIN_SECRETION
HALLMARK_MYC_TARGETS_V2
HALLMARK_ADIPOGENESIS
HALLMARK_BILE_ACID_METABOLISM
HALLMARK_INFLAMMATORY_RESPONSE
HALLMARK_APOPTOSIS
HALLMARK_INTERFERON_ALPHA_RESPONSE
HALLMARK_IL6_JAK_STAT3_SIGNALING
WP_PROSTAGLANDIN_SIGNALING
KEGG_ALLOGRAFT_REJECTION
HALLMARK_G2M_CHECKPOINT
KEGG_GRAFT_VERSUS_HOST_DISEASE
WP_TYPE_II_INTERFERON_SIGNALING_IFNG
HALLMARK_ALLOGRAFT_REJECTION
KEGG_RIBOSOME
WP_CYTOPLASMIC_RIBOSOMAL_PROTEINS
HALLMARK_INTERFERON_GAMMA_RESPONSE
HALLMARK_TNFA_SIGNALING_VIA_NFKB
Pathway
NES
Cluster 2: TAM_Proangiogenic
HALLMARK_HYPOXIA
WP_CCL18_SIGNALING_PATHWAY
KEGG_FRUCTOSE_AND_MANNOSE_METABOLISM
WP_GLUCOCORTICOID_RECEPTOR_PATHWAY
HALLMARK_PROTEIN_SECRETION
HALLMARK_APOPTOSIS
WP_NRF2_PATHWAY
WP_OREXIN_RECEPTOR_PATHWAY
HALLMARK_P53_PATHWAY
WP_PROXIMAL_TUBULE_TRANSPORT
HALLMARK_TGF_BETA_SIGNALING
WP_VEGFAVEGFR2_SIGNALING_PATHWAY
HALLMARK_ANGIOGENESIS
HALLMARK_MITOTIC_SPINDLE
HALLMARK_MYC_TARGETS_V1
WP_TCA_CYCLE_AKA_KREBS_OR_CITRIC_ACID_CYCLE
WP_TRYPTOPHAN_METABOLISM
WP_ATM_SIGNALING_IN_DEVELOPMENT_AND_DISEASE
KEGG_CITRATE_CYCLE_TCA_CYCLE
WP_DNA_REPLICATION
WP_DNA_REPAIR_PATHWAYS_FULL_NETWORK
WP_CHOLESTEROL_BIOSYNTHESIS_PATHWAY
HALLMARK_MYC_TARGETS_V2
KEGG_DNA_REPLICATION
HALLMARK_G2M_CHECKPOINT
HALLMARK_E2F_TARGETS
Pathway
NES
Cluster 6: TAM_Immune regulatory
KEGG_OXIDATIVE_PHOSPHORYLATION
HALLMARK_OXIDATIVE_PHOSPHORYLATION
HALLMARK_COAGULATION
HALLMARK_PROTEIN_SECRETION
HALLMARK_HYPOXIA
WP_ELECTRON_TRANSPORT_CHAIN_OXPHOS_SYSTEM_IN_MITOCHONDRIA
KEGG_LYSOSOME
WP_OXIDATIVE_PHOSPHORYLATION
WP_MITOCHONDRIAL_COMPLEX_IV_ASSEMBLY
WP_BURN_WOUND_HEALING
HALLMARK_ANGIOGENESIS
HALLMARK_MITOTIC_SPINDLE
HALLMARK_INTERFERON_GAMMA_RESPONSE
WP_ALLOGRAFT_REJECTION
WP_DNA_REPLICATION
WP_CYTOPLASMIC_RIBOSOMAL_PROTEINS
HALLMARK_INTERFERON_ALPHA_RESPONSE
KEGG_ALLOGRAFT_REJECTION
WP_CANCER_IMMUNOTHERAPY_BY_PD1_BLOCKADE
WP_TYPE_II_INTERFERON_SIGNALING_IFNG
HALLMARK_G2M_CHECKPOINT
HALLMARK_E2F_TARGETS
Pathway
NES
Cluster 14: TAM_Proliferating
HALLMARK_G2M_CHECKPOINT
HALLMARK_E2F_TARGETS
HALLMARK_MITOTIC_SPINDLE
KEGG_CELL_CYCLE
WP_CELL_CYCLE
HALLMARK_SPERMATOGENESIS
KEGG_LYSOSOME
WP_MITOCHONDRIAL_COMPLEX_I_ASSEMBLY_MODEL_OXPHOS_SYSTEM
WP_OXIDATIVE_PHOSPHORYLATION
WP_TCA_CYCLE_AKA_KREBS_OR_CITRIC_ACID_CYCLE
HALLMARK_OXIDATIVE_PHOSPHORYLATION
WP_TYPE_II_INTERFERON_SIGNALING_IFNG
WP_MEASLES_VIRUS_INFECTION
KEGG_JAK_STAT_SIGNALING_PATHWAY
HALLMARK_INTERFERON_ALPHA_RESPONSE
WP_ONCOSTATIN_M_SIGNALING_PATHWAY
KEGG_LEISHMANIA_INFECTION
HALLMARK_ALLOGRAFT_REJECTION
HALLMARK_HYPOXIA
HALLMARK_INTERFERON_GAMMA_RESPONSE
HALLMARK_TNFA_SIGNALING_VIA_NFKB
KEGG_RIBOSOME
WP_CYTOPLASMIC_RIBOSOMAL_PROTEINS
Pathway
NES

Figure S3, Related to Figure 3. Gene expression and pathway enrichment analysis on macrophage populations

(A) Heatmap of top 5 differentially expressed genes in each cluster.

(B) Pathway enrichment analysis of macrophage clusters by GSEA. The colored pathways indicate relevance to the annotated function of the cluster.

(C) UMAP plots of sub-clustering analysis of cluster 7.

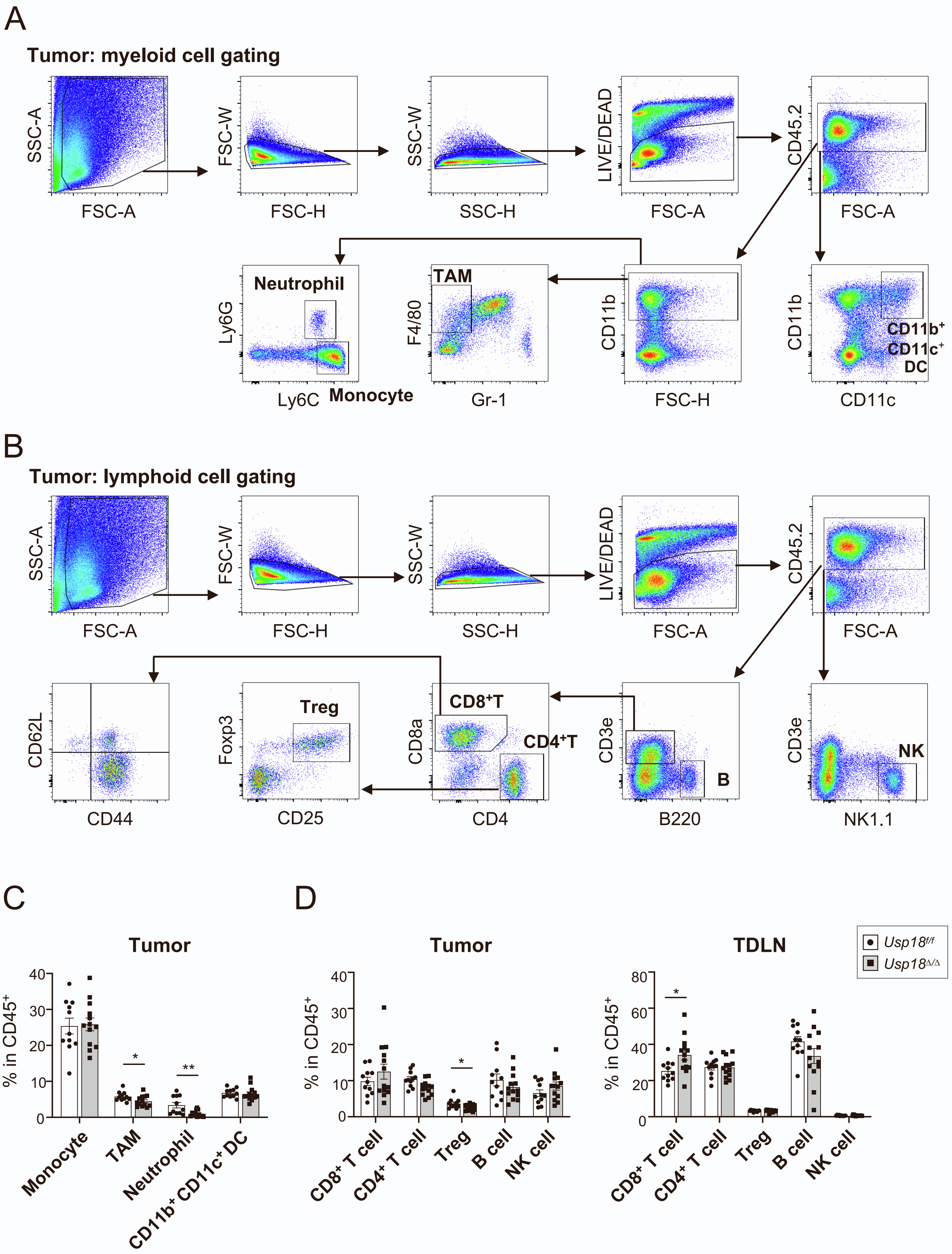
A
Tumor: myeloid cell gating
SSC-A
FSC-A
FSC-W
FSC-H
SSC-W
SSC-H
LIVE/DEAD
FSC-A
CD45.2
FSC-A
Neutrophil
Ly6G
Ly6C
Monocyte
TAM
F4/80
Gr-1
CD11b
FSC-H
CD11b
CD11c
CD11b+
CD11c+
DC
B
Tumor: lymphoid cell gating
SSC-A
FSC-A
FSC-W
FSC-H
SSC-W
SSC-H
LIVE/DEAD
FSC-A
CD45.2
FSC-A
CD62L
CD44
Treg
Foxp3
CD25
CD8+T
CD4+T
CD8a
CD4
CD3e
B220
B
CD3e
NK
NK1.1
C
Tumor
% in CD45+
Monocyte
TAM
Neutrophil
CD11b+ CD11c+ DC
D
Tumor
% in CD45+
CD8+ T cell
CD4+ T cell
Treg
B cell
NK cell
TDLN
% in CD45+
CD8+ T cell
CD4+ T cell
Treg
B cell
NK cell
Usp18f/f
Usp18Δ/Δ

Figure S4, Related to Figure 4. Flow cytometry analysis of immune cells in tumor and tumor-draining lymph node

(A, B) Gating strategy for subsets of immune cells by flow cytometry; myeloid subsets (A) and lymphoid subsets (B). Samples from tumor are shown.

(C) Percentages of myeloid subsets in tumor.

(D) Percentage of lymphoid subsets in tumor and tumor-draining lymph node (TDLN).

Usp18$^{f/f}$ (n = 11), *Usp18*$^{\Delta/\Delta}$ (n = 13). Mean ± S.E.M., two-tailed unpaired *t*-test, *; $p < 0.05$, **; $p < 0.01$
